# Ligand Profiling to Characterize Different Polymorphic Forms of α-Synuclein Aggregates

**DOI:** 10.1021/jacs.3c10521

**Published:** 2023-11-29

**Authors:** Timothy S. Chisholm, Christopher A. Hunter

**Affiliations:** Yusuf Hamied Department of Chemistry, University of Cambridge, Lensfield Road, Cambridge CB2 1EW, U.K.

## Abstract

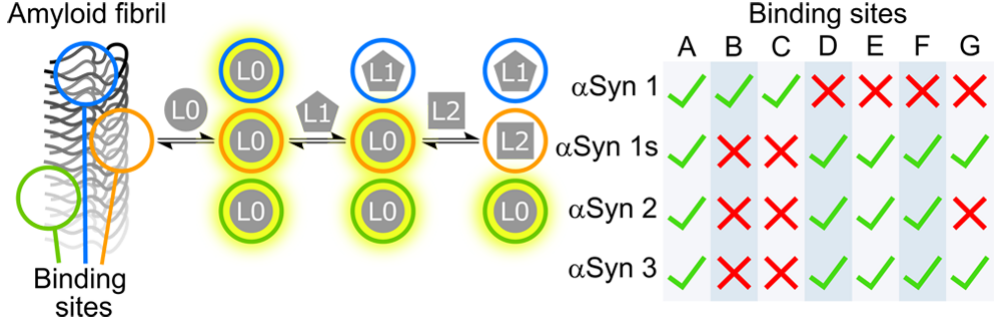

The presence of amyloid fibrils is a characteristic feature of many diseases, most famously neurodegenerative disease. The supramolecular structure of these fibrils appears to be disease-specific. Identifying the unique morphologies of amyloid fibrils could, therefore, form the basis of a diagnostic tool. Here we report a method to characterize the morphology of α-synuclein (αSyn) fibrils based on profiling multiple different ligand binding sites that are present on the surfaces of fibrils. By employing various competition binding assays, seven different types of binding sites were identified on four different morphologies of αSyn fibrils. Similar binding sites on different fibrils were shown to bind ligands with significantly different affinities. We combined this information to construct individual profiles for different αSyn fibrils based on the distribution of binding sites and ligand interactions. These results demonstrate that ligand-based profiling can be used as an analytical method to characterize fibril morphologies with operationally simple fluorescence binding assays.

## Introduction

Neurodegenerative diseases, such as Alzheimer’s and Parkinson’s, are one of the foremost health challenges faced in contemporary society.^1−5^ These diseases are characterized by the deposition of insoluble protein aggregates within the brain.^6−8^ In Parkinson’s disease, these protein aggregates are composed of α-synuclein (αSyn) fibrils. αSyn itself is a 140-residue, intrinsically disordered protein with a native function that is not yet fully understood. However, it is believed that αSyn plays a role in synapse function and plasticity, and in regulating synaptic vesicle release and trafficking.^9−11^ The formation of fibrillar αSyn assemblies within nerve cells is a defining feature of Parkinson’s disease (PD), multiple system atrophy (MSA), and dementia with Lewy bodies (DLB), and is often used as a post-mortem hallmark for disease diagnosis.^12−14^ Collectively, these disorders are referred to as synucleinopathies. In PD and DLB, aggregated αSyn is deposited in round, dense neuronal inclusions named Lewy bodies, and within abnormal neurites termed Lewy neurites.^15−22^ Within MSA, αSyn aggregates are predominantly found within oligodendroglia as glial cytoplasmic inclusions.^23^

While the precise mechanism of αSyn aggregation remains unclear, several high-resolution cryo-electron microscopy structures of αSyn fibrils have been determined.^24−31^ For both αSyn and other amyloidogenic proteins, the structures of fibrils formed in vitro are strongly dependent on the aggregation conditions used.^32−36^ Additionally, the structures of fibrils formed in vivo appear to be disease-specific.^37^ Relating the underlying disease to the morphology of αSyn fibrils could therefore form the basis of a diagnostic method.

Protein fibrils possess multiple structurally distinct binding sites that bind different structural classes of ligands.^38−49^ The nature of the binding sites and their spatial distribution differs between fibrils composed of different proteins.^50^ Characterization of the ligand binding sites present on amyloid fibrils may therefore provide a methodology for fingerprinting fibril morphology. Here we show that it is possible to characterize four morphologically distinct types of αSyn fibrils by characterizing differences in ligand binding sites.

## APPROACH

**Figure 1:**
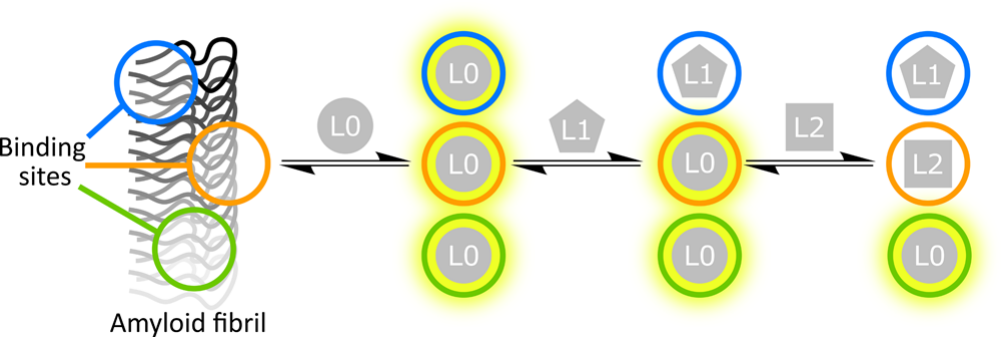
A two-step competition binding assay showing the binding of three ligands (L0, L1, and L2) to fibril binding sites (blue, orange, and green circles). First, the solvatochromic ligand L0 is added to a sample of fibrils. L0 binds to all three sites, producing an enhancement in fluorescence emission intensity. A competition assay is then performed by titrating in the competing ligand L1 which displaces L0 from one binding site (blue), producing a change in fluorescence. A second competition binding assay is then performed using another competing ligand L2 which displaces L0 from a second site (orange).

Two-step fluorescence competition binding assays provide a powerful tool for interrogating the distribution of binding sites present on amyloid fibrils by using multiple ligands. Figure 1 illustrates the approach. First, the fluorescent solvatochromic ligand L0 is titrated into a sample of fibrils, filling all of the accessible binding sites. Next, a nonfluorescent ligand (L1) is titrated into the same sample, and binding of L1 is monitored via the change in fluorescence associated with displacement of L0. If all of the bound L0 is displaced in this experiment, then we conclude that all of the L0 sites are accessible to L1. If L0 is only partially displaced, we conclude that there is at least one binding site that is accessible to L0 but not to L1. In this case, a second nonfluorescent ligand (L2) can be titrated into the mixture of fibrils, L0, and L1, and any further change in fluorescence can be used to monitor binding of L2. If L2 displaces L0 in this experiment, we conclude that there is a binding site that is accessible to L2 and to L0, but not to L1. Two-step competition binding assays can, therefore, be used to reveal the presence of multiple different binding sites. This assay format also allows quantification of the binding of ligands to different subsets of binding sites, and measurements using multiple combinations of ligands can therefore provide a detailed profile of the binding site landscapes present on different fibril morphologies.

## RESULTS and DISCUSSION

### Preparation of Ligands

**Figure 2:**
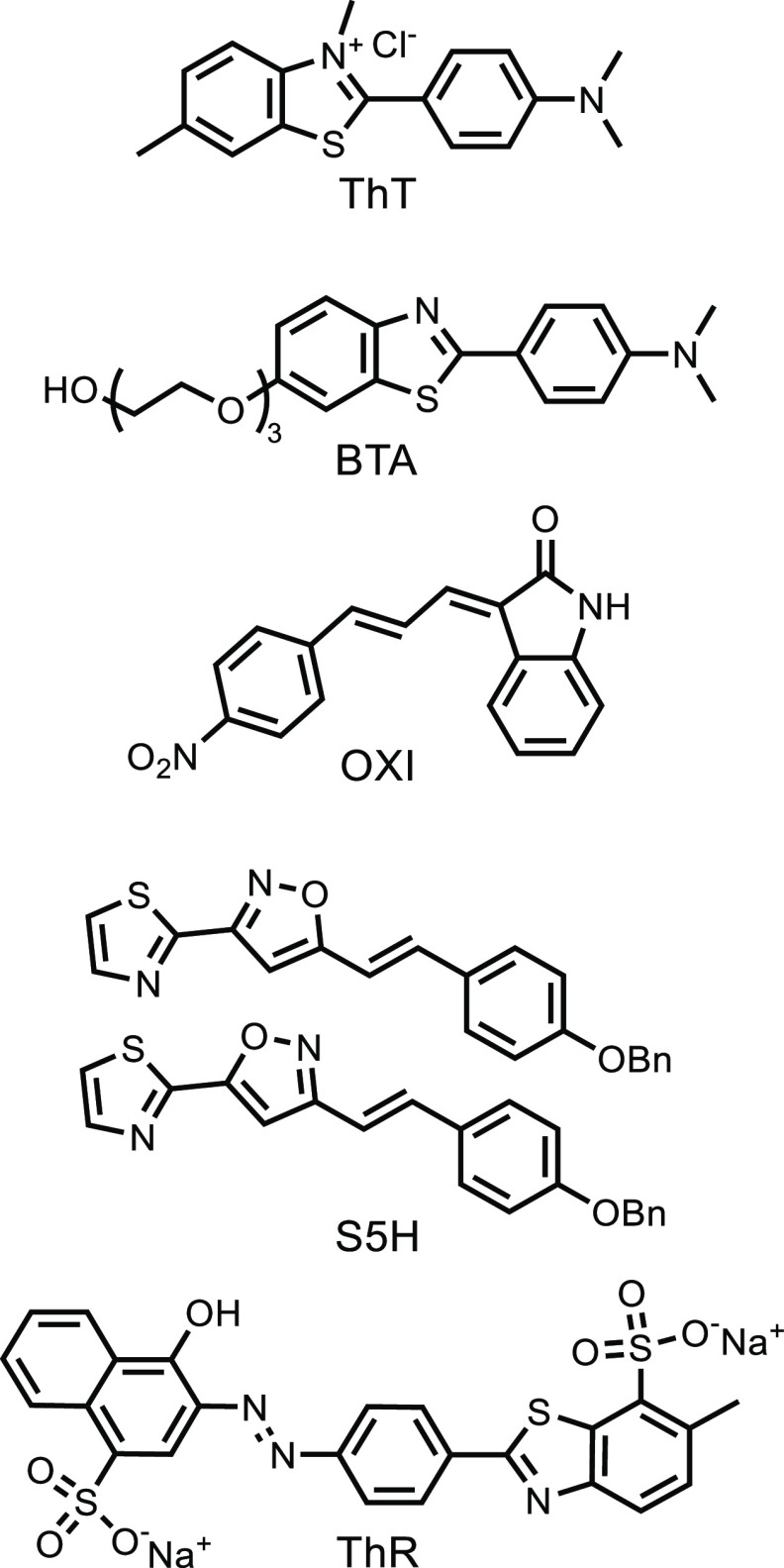
Chemical structures of the ligands used in binding assays.

Five ligands were used in the binding assays (Figure 2). Thioflavin T (ThT) was chosen as the primary fluorescent ligand used to monitor binding (L0). ThT is widely used, readily available, and solvatochromic with a large change in fluorescence properties on binding to αSyn fibrils. Commercial samples of ThT were recrystallized prior to use to ensure purity. The neutral benzothiazole derivative BTA was prepared by a literature procedure, followed by coupling to an ethylene glycol oligomer to enhance solubility.^51^ BTA is fluorescent but is not solvatochromic. Three nonfluorescent ligands were then selected to use in competition assays. Thiazine red (ThR) is a commercially available ligand with negatively charged sulfonate groups. These negatively charged groups were hypothesized to direct ThR to different binding sites than the positively charged ThT. The oxindole ligand OXI and the styryl-oxazole ligand S5H were synthesized based on previous work.^52,53^ S5H was obtained and used as a mixture of the 3,5-isoxazole and 5,3-isoxazole isomers, as previously reported.

### Characterization of Fibrils

**Figure 3:**
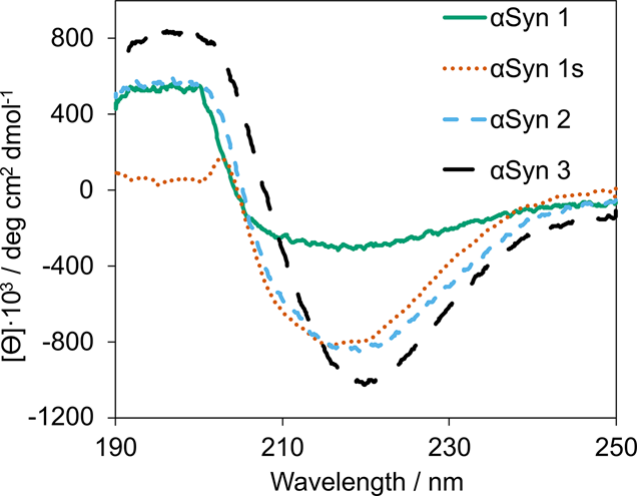
Circular dichroism spectra of αSyn fibrils (1.0 μM) recorded in aqueous 1xPBS buffer (pH 7.4, 25 °C).

**Figure 4:**
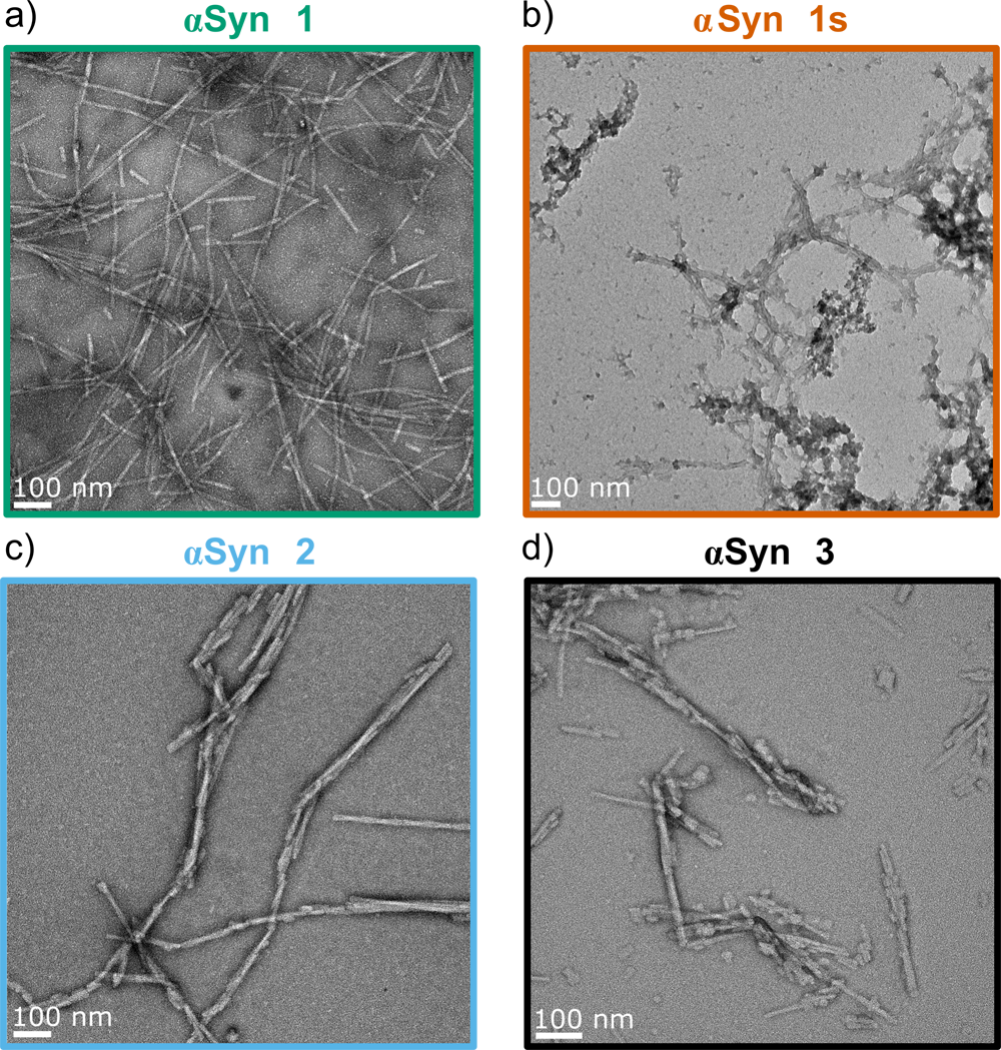
Transmission electron microscopy images of the different αSyn fibrils: a) αSyn 1, b) αSyn 1s, c) αSyn 2, d) αSyn 3.

Four morphologically distinct preparations of αSyn fibrils (denoted as αSyn 1, αSyn 1s, αSyn 2, and αSyn 3) were obtained by aggregation under different conditions. Cryo-EM studies have shown that fibrils formed in buffers containing phosphate are morphology distinct from fibrils formed in Tris buffer.^24−26,54^ Acidic buffers also influence fibril morphology.^36^ αSyn 1 was prepared in 1xPBS buffer (pH 7.4, 37 °C, 72 h). αSyn 1s was prepared in 1xPBS buffer (pH 7.4, 37 °C, 72 h) and then sonicated and stored at −20 °C for 6 months. αSyn 2 was prepared in Tris buffer (50 mM, 100 mM NaCl, pH 7.5, 37 °C, 72 h), and αSyn 3 was prepared in MES buffer (20 mM, pH 6.1, 37 °C, 72 h). The circular dichroism spectra of each fibril sample showed a minimum at 220 nm, which is characteristic of β-sheet aggregates (Figure 3). Transmission electron microscopy images show differences in the length of the fibrils, the degree of lateral association between fibrils, and the heterogeneity of the sample (Figure 4), indicating that the four αSyn fibril preparations are morphologically distinct.

### Saturation Binding Assays

**Figure 5:**
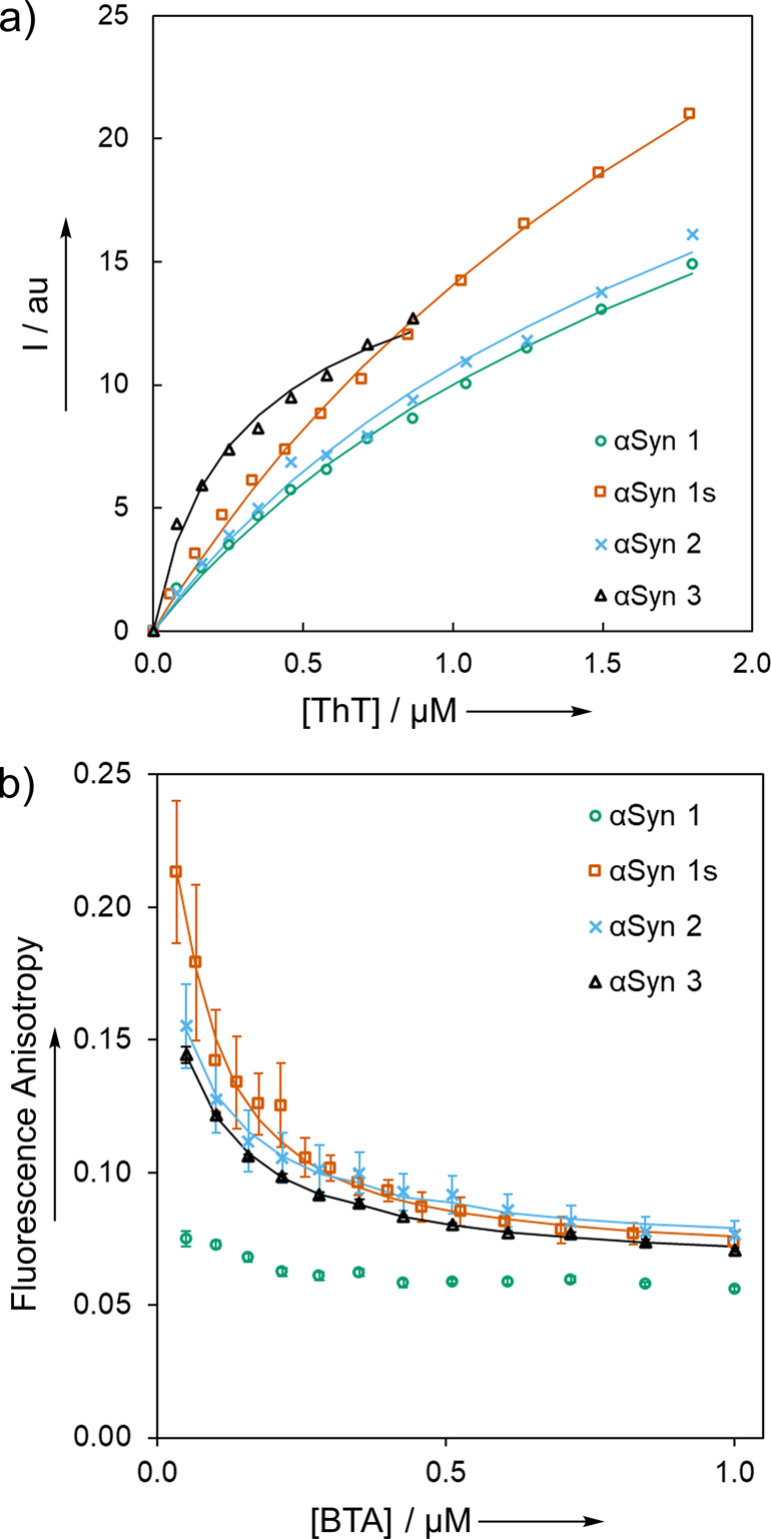
a) Data from fluorescence titrations of a) ThT (λ_ex_ = 440 nm, λ_em_ = 483 nm), and b) BTA (λ_ex_ = 360 nm, λ_em_ = 443 nm), into αSyn fibrils (500 nM) in aqueous 1xPBS (pH 7.4, 25 °C). Data points are the average of at least three experimental measurements with 95% confidence intervals shown, and lines are the best fit to a 1:1 binding isotherm.

The αSyn fibrils were first studied by using ThT fluorescence saturation binding assays. Titrating ThT into a solution of αSyn fibrils (500 nM) in 1xPBS buffer (pH 7.4, 25 °C) yielded an enhancement in fluorescence intensity characteristic of binding to a β-sheet aggregate (Figure 5a).^55^ Fitting a 1:1 binding isotherm to these data showed that ThT binds to αSyn 1, αSyn 1s, and αSyn 2 with a similar affinity (−log(*K*_d_/M) = 5.7–5.9), where *K*_d_ is the dissocation constant. However, the binding affinity for αSyn 3 is significantly higher (−log(*K*_d_/M) = 6.7).

The fluorescence spectrum of BTA did not show a significant change upon binding to αSyn fibrils, but the fluorescence anisotropy of BTA proved to be a useful probe of binding. Fluorescence anisotropy data for titration of BTA into αSyn fibrils (500 nM, 1xPBS, pH 7.4, 25 °C) are shown in Figure 5b. Fitting a 1:1 binding isotherm to these data showed that BTA binds to αSyn 1s, αSyn 2, and αSyn 3 with nanomolar affinity (−log(*K*_d_/M) = 7.1–7.8), but negligible binding was observed for αSyn 1.

### One-Step Competition Binding Assays

**Figure 6:**
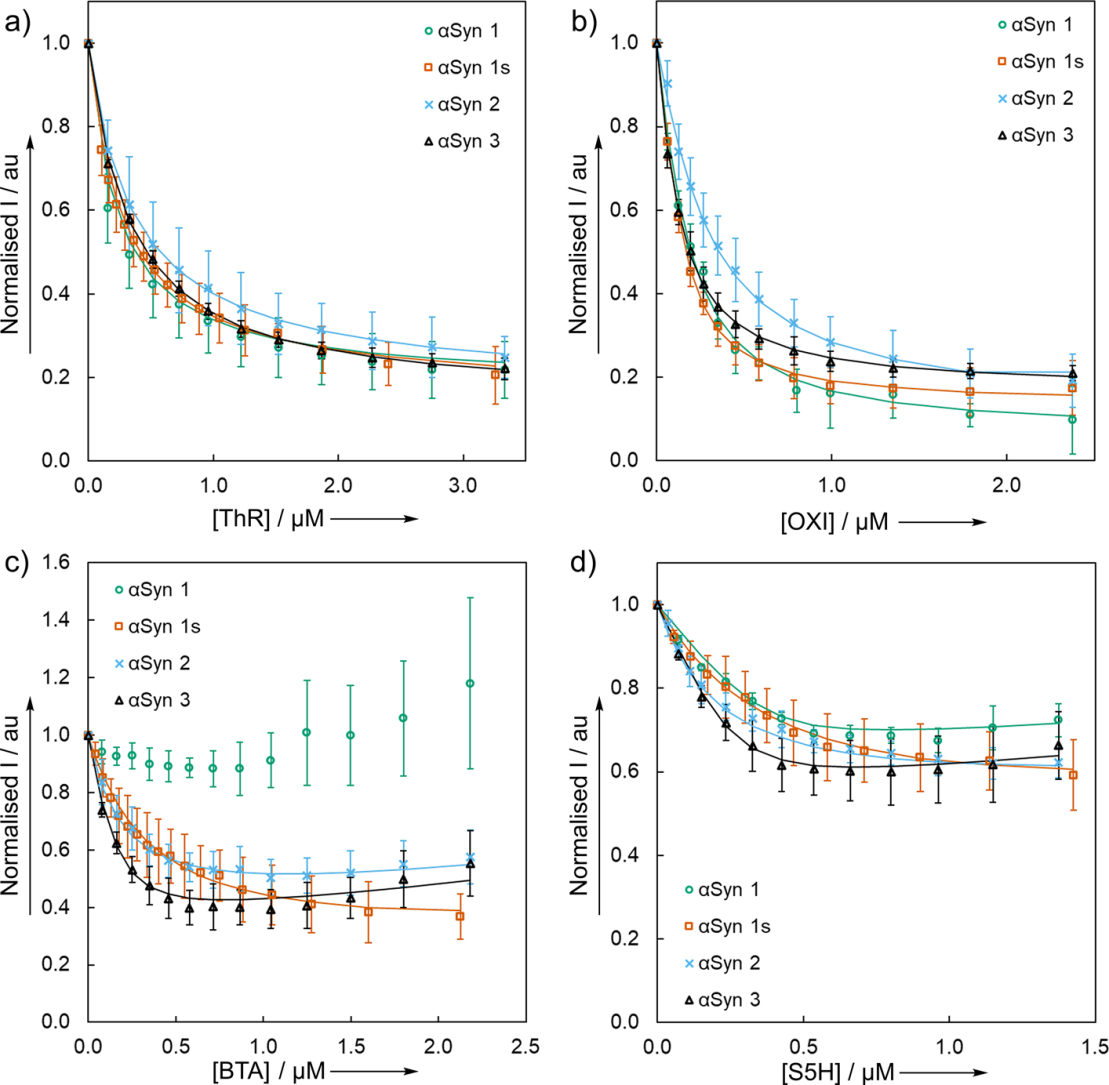
Fluorescence competition assays for titration of a) ThR, b) OXI, c) BTA, and d) S5H into a mixture of αSyn fibrils (500 nM) and ThT (1.0 μM, λ_ex_ = 440 nm, λ_em_ = 483 nm) in aqueous 1xPBS buffer (pH 7.4, 25 °C). Data points are the average of at least three experimental measurements with 95% confidence intervals shown, and lines are the best fit to a 1:1 binding isotherm.

One-step fluorescence competition assays were then performed to study the binding of nonfluorescent ligands to αSyn fibrils. This assay format corresponds to the second step in Figure 1, showing the displacement of L0 by L1. The ligands BTA, OXI, S5H, and ThR (L1) were titrated into a solution of αSyn fibrils (500 nM) and ThT (1.0 μM, L0) in 1xPBS buffer (pH 7.4, 25 °C). BTA and S5H are weakly fluorescent at the wavelength used to excite ThT (λ_ex_ = 440 nm), and this is accounted for during data fitting, whereas ThR and OXI are negligibly fluorescent (see SI). The titration data were fitted to 1:1 binding isotherms to obtain the dissociation constant for L1 using the known dissociation constant for L0 (Figure 6, see SI for details). As the change in ThT fluorescence was monitored, these assays report only the binding of L1 to ThT binding sites.

#### ThR and OXI

Both ThR and OXI displaced a similar amount of ThT (Figure 6a and 6b). These two ligands therefore appear to share a similar set of ThT-binding sites on all four αSyn fibrils. At the end of the titration, there was always a residual ThT fluorescence signal. This residual fluorescence suggests that there is an additional ThT-binding site on these fibrils that neither OXI nor ThR can access.

#### BTA

The titration data in Figure 6c appear slightly different due to the fluorescence of BTA, which increases during the titration. However, BTA displaced a similar proportion of ThT as ThR and OXI for αSyn 1s, αSyn 2, and αSyn 3. BTA did not displace ThT from αSyn 1 fibrils, which is in line with the lack of binding observed in the fluorescence anisotropy experiment shown in Figure 5b. These results suggest that BTA may share the same binding site as ThR and OXI on all fibrils except αSyn 1.

#### S5H

S5H is also weakly fluorescent, so there was a slight increase in fluorescence intensity toward the end of these titrations (Figure 6d). S5H displaced a similar proportion of ThT from all four fibril morphologies. However, S5H displaced less ThT than OXI, ThR, or BTA. This result suggests that ThR, OXI, and BTA bind to two different sites, only one of which is accessible to S5H.

### One-Step Blocked Site Assays

**Figure 7:**
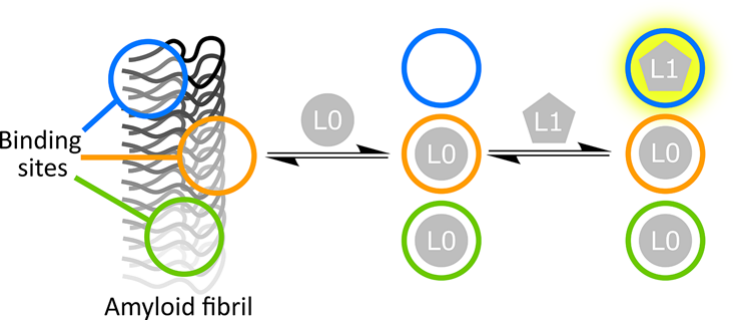
A one-step blocked site assay showing the binding of two different ligands (L0 and L1) to different fibril binding sites (blue, orange, and green circles). First, a nonfluorescent ligand L0 is added to a sample of fibrils, and then a solvatochromic ligand L1 is added. If L0 has a greater affinity than L1, L1 will only bind to sites that are not occupied by L0.

A variation of the one-step competition assay is the one-step blocked site assay shown in Figure 7. In this case, a subset of binding sites is first blocked by a high-affinity, nonfluorescent ligand L0, and then a lower affinity fluorescent ligand L1 is added. L1 will only be able to bind to sites that are not occupied by L0. αSyn fibrils (500 nM) were combined with ThR, OXI, or S5H (1.4–5.0 μM, L0) in 1xPBS buffer (pH 7.4, 25 °C), and then ThT (L1) was titrated into this solution. The change in ThT fluorescence was monitored and fitted to a 1:1 binding isotherm. The resulting dissociation constant for binding of ThT was similar for all of the L0 ligands and for all of the αSyn fibrils (−log(*K*_d_/M) = 5.5–6.1).

### Two-Step Competition Binding Assays

**Figure 8:**
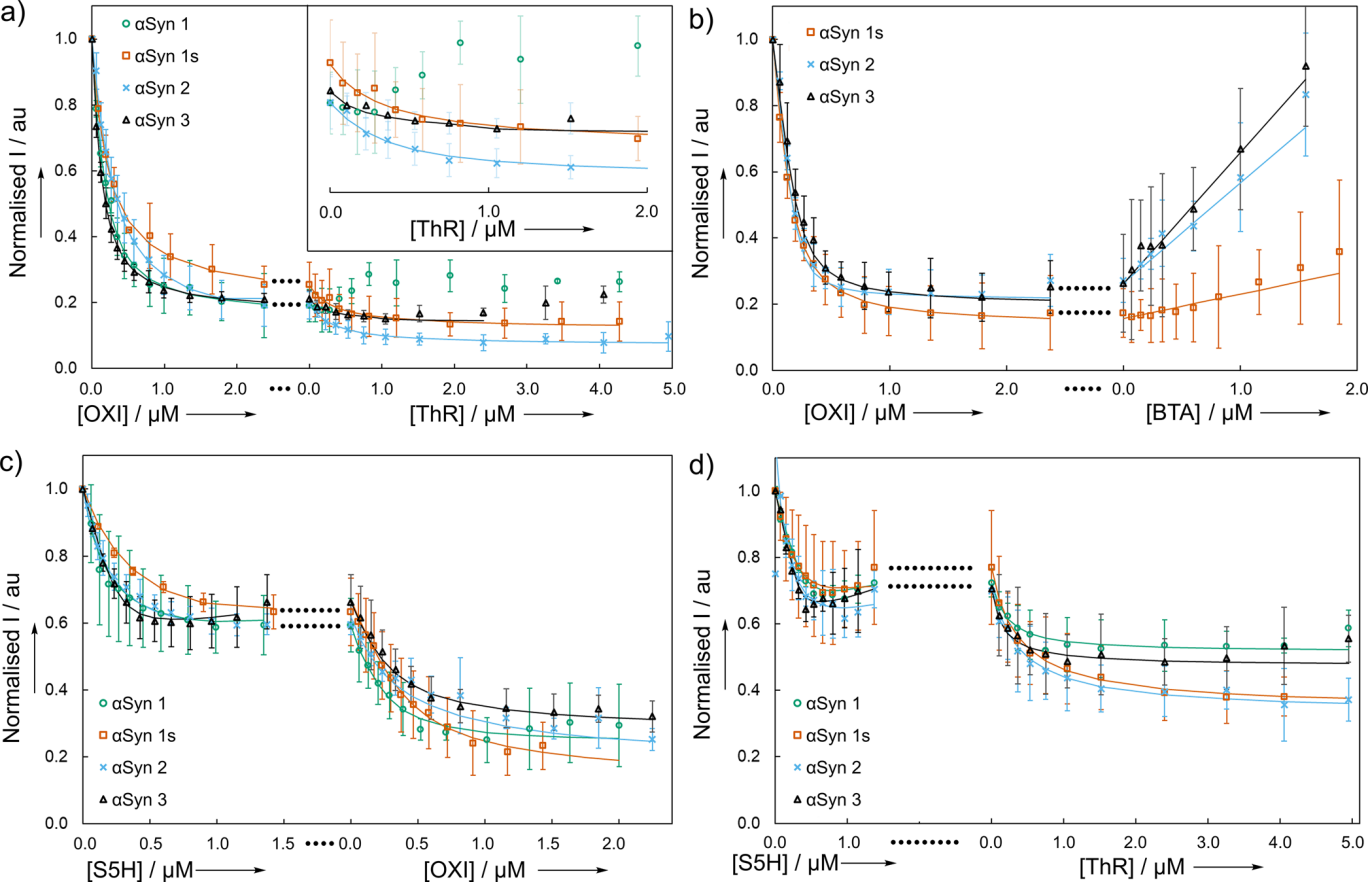
Two-step competition assays for the sequential titration of two ligands into αSyn fibrils (500 nM) and ThT (L0, 1.0 μM, λ_ex_ = 440 nm, λ_em_ = 483 nm) in aqueous 1xPBS buffer (pH 7.4, 25 °C). The ligands used are a) OXI (L1) and then ThR (L2) with an expansion of the addition of ThR, b) OXI (L1) and then BTA (L2), c) S5H (L1) and then OXI (L2), and d) S5H (L1) and then ThR (L2). The second phase of the two-step competition assays is plotted continuously after the first phase, and the horizontal lines indicate the change in ligand. Data points are the average of at least three experimental measurements with 95% confidence intervals shown, and lines are the best fit to 1:1 binding isotherms.

Two-step competition binding assays were then performed to better interrogate the presence of multiple binding sites on each αSyn fibril. In the notation of Figure 1, a one-step competition binding assay was first performed involving displacement of ligand L0 with ligand L1. Next, ligand L2 was titrated into the mixture. If L2 displaces additional L0, then L2 must share a binding site with L0 that is not accessible to L1. The results of two-step competition binding assays using ThT as the L0 ligand are shown in Figure 8.

#### OXI (L1) then ThR (L2)

ThR (L2) showed a small amount of additional displacement of ThT (L0) for αSyn 1s, αSyn 2, and αSyn 3, but not for αSyn 1 (Figure 8a). This result suggests that there is a binding site that is accessible to ThR and ThT, but not to OXI on αSyn 1s, αSyn 2, and αSyn 3 fibrils.

#### OXI (L1) then BTA (L2)

BTA (L2) showed no additional displacement of ThT (L0) (Figure 8b), indicating that OXI and BTA share the same subset of ThT-binding sites on αSyn 1s, αSyn 2, and αSyn 3. This assay was not performed with αSyn 1, since BTA does not bind to this fibril.

#### S5H (L1) then OXI (L2)

OXI (L2) displaced additional ThT (L0) from all four fibrils (Figure 8c). The final proportion of ThT displaced was similar to that of the one-step competition assay with OXI shown in Figure 6b, which suggests that all of the S5H sites are accessible to OXI, but there is an OXI site that is not accessible to S5H.

#### S5H (L1) then ThR (L2)

ThR (L2) displaced additional ThT (L0) from all four fibrils (Figure 8d). The final proportion of ThT displaced was comparable to that of the one-step competition assay with ThR shown in Figure 6a, which suggests that all of the S5H sites are accessible to ThR, but there is a ThR site that is not accessible to S5H.

### Identification of Different Binding Sites

**Figure 9:**
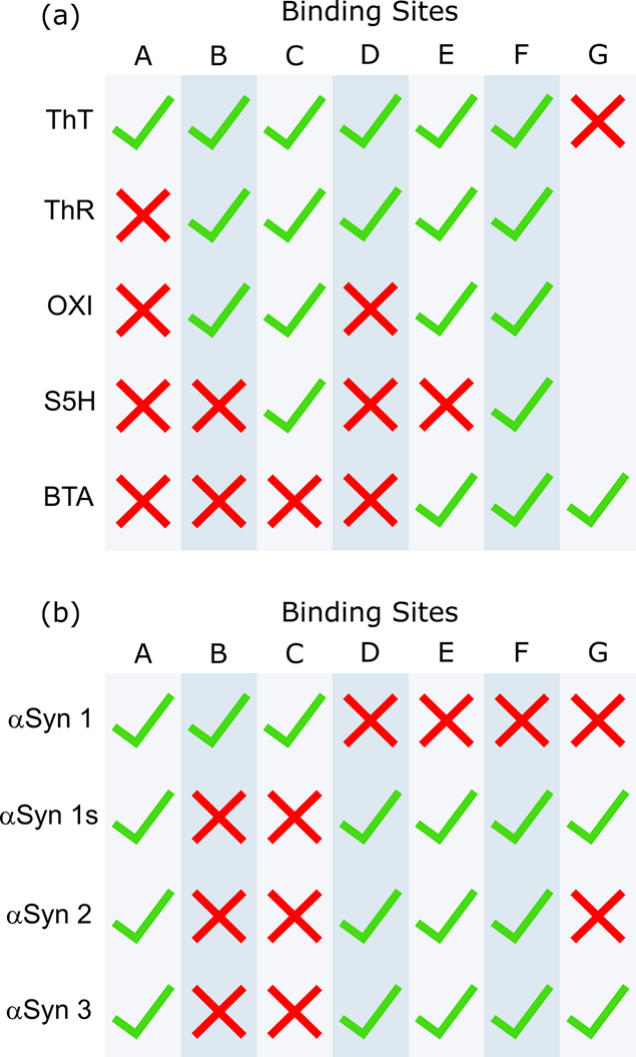
a) Types of binding sites (labeled A–G) identified on αSyn fibrils and the ligands that target each of these sites. Ticks and crosses indicate whether a ligand does or does not bind to a site. A blank entry indicates it is unknown whether a ligand targets that site. b) The binding site profile of different αSyn fibril polymorphs. Ticks and crosses indicate the presence or absence of a binding site on the fibril.

Using the results of these binding assays, six different types of binding sites were identified on the αSyn fibrils studied. Binding sites are defined in terms of the ligands that bind to that site based on ThT competition assays, so only sites that are also accessible to ThT are identified. Figure 9a summarizes the six binding sites identified, and Figure 9b shows the distribution of these binding sites that are present on each αSyn fibril.

None of the ligand combinations completely displaced ThT from any αSyn fibril. This means that all fibrils must have a ThT binding site that does not bind any of the other four ligands studied. This ThT-only site is designated as Site A (Figure 9a).

αSyn 1 did not bind BTA, meaning these fibrils have a unique set of binding sites. The competition assays with ThR and OXI show that they displace the same amount of ThT from αSyn 1 fibrils (Figure 6a, 6b, 8a), which suggests that these ligands access the same binding sites. The competition assays with S5H show that this ligand displaced less ThT from αSyn 1 fibrils than OXI and ThR (Figure 6, 8c, 8d), so there must be a site on αSyn 1 that binds ThR and OXI but not S5H. The site on αSyn 1 fibrils that binds ThR and OXI, but not S5H is designated Site B, and the site that binds all three of ThR, OXI, and S5H is designated Site C (Figure 9a).

For αSyn 1s, αSyn 2, and αSyn 3 fibrils, two-step competition assays showed that ThR (L2) displaced more ThT than OXI (L1) (Figure 8a), which suggests that there is a site that is accessible to ThR but not to OXI. This site is designated Site D (Figure 9a). Two-step competition assays show that BTA binds to the same sites as OXI (Figure 8b) and S5H binds to a subset of the OXI sites (Figure 8c, 8d). Since OXI cannot access Site D, neither do BTA or S5H. The site that binds ThR, OXI, and BTA is designated Site E, and the site that binds all of the ligands is designated Site F (Figure 9a).

Figure 9 shows that there is a relatively complex distribution of different binding sites present on αSyn fibrils. A total of six binding sites were identified, with each accessed by a different set of ligands. Three of the fibrils, αSyn 1s, αSyn 2, and αSyn 3, have the same combination of four binding sites (A, D, E, and F). αSyn 1 has a different combination of three binding sites (A, B, and C) and does not bind BTA. This complexity suggests that ligand competition assays may be analytically useful for identifying and characterizing amyloid fibrils. For example, identification of the presence of ligand binding sites B or C could be used to distinguish αSyn 1 fibrils from the others.

### Quantitative Measurements

The dissociation constant and the relative concentrations of different binding sites were determined by fitting the titration data to the relevant binding isotherm. Using the binding site model outlined in Figure 9, the binding sites that each assay reports on can be determined (see SI for details). For example, titrating ThT into a solution of αSyn 1 (L0 = ThT, L1 = L2 = none) reports on ThT binding to Sites A, B, and C. However, titrating ThT into a solution of αSyn 1 and OXI (L0 = ThT, L1 = OXI, L2 = none) reports only on Site A as OXI is already bound to Site B and C. The dissociation constants for binding of ThT are similar for each binding site and each type of fibril (−log(*K*_d_/M) = 5.5–6.1). There is one outlier that comes from direct titration of ThT into αSyn 3 (−log(*K*_d_/M) = 6.7). This experiment targets Sites A, D, E, and F. The other titrations with αSyn 3 target sites A, D, and E, which suggests that it is Site F on αSyn 3 that is the high-affinity site for ThT.

The dissociation constants for BTA are slightly higher (−log(*K*_d_/M) = 6.6–7.2, see SI for details). For αSyn 1s and αSyn 3 fibrils, the dissociation constants measured by a direct fluorescence anisotropy titration and by fluorescence competition with ThT as L0 differ by an order of magnitude, which suggests that there is an additional seventh site on these fibrils that binds BTA but not ThT. This site is designated Site G. BTA has a high affinity for Site G in αSyn 1s (−log(*K*_d_/M) = 7.8), and a higher affinity for Sites E and/or F in αSyn 3 (−log(*K*_d_/M) = 8.0).

The dissociation constants measured for OXI by competition assays are lower for αSyn 1 and αSyn 1s (−log(*K*_d_/M) = 7.2–7.4) than for αSyn 2 and αSyn 3 (−log(*K*_d_/M) = 8.0–8.1), which indicates that OXI binds more strongly to Site F on αSyn 2 and αSyn 3.

The dissociation constants measured for S5H all have similar values (−log(*K*_d_/M) = 6.7–7.3).

ThR has a higher affinity for αSyn 3 (−log(*K*_d_/M) = 7.5) than for the other fibrils (−log(*K*_d_/M) = 6.7–7.0). Although Sites D–F are all implicated, the high affinity site on αSyn 3 is most likely Site D.

The relative concentrations of ligand binding sites on αSyn fibrils are summarized in Tables 1 and 2. In order to fit the fluorescence competition titration data, the populations of three different sites were optimized: one site accessible only to ThT (L0), one site accessible to L0 and L1, and one site accessible to L0 and L2 but not to L1. The proportions of different sites present could therefore be determined from the limiting amount of ThT displaced by each of the competing ligands, assuming that the optical brightness of bound ThT is equivalent for all binding sites. The one-step competition data in Table 1 show that S5H occupies 40–60% of ThT binding sites on all of the fibrils, whereas OXI, BTA, and ThR occupy approximately 80% of the ThT sites. As above, the exception is BTA, which does not bind to αSyn 1. Two-step competition data in Table 2 show that, for all of the fibrils, 20–30% of ThT binding sites are accessible to ThR and OXI but not to S5H. For all fibrils except αSyn 1, approximately 10% of ThT binding sites are accessible to ThR but not to OXI.

**Table 1:** Percentage of ThT Binding Sites Accessed by L1^a^

		Fibril			
L0	L1	αSyn 1	αSyn 1s	αSyn 2	αSyn 3
ThT	S5H	40%	60%	50%	50%
ThT	OXI	90%	80%	80%	80%
ThT	ThR	80%	80%	80%	80%
ThT	BTA	0%	90%	70%	70%

a Percentages are the average of at least three independent experiments reported to one significant figure.

**Table 2:** Percentage of ThT Binding Sites Accessed by L2 but Not L1^a^

			Fibril			
L0	L1	L2	αSyn 1	αSyn 1s	αSyn 2	αSyn 3
ThT	S5H	OXI	30%	30%	30%	30%
ThT	S5H	ThR	20%	20%	20%	20%
ThT	OXI	ThR	0%	10%	10%	10%
ThT	OXI	BTA	0%	0%	0%	0%

a Percentages are the average of at least three independent experiments reported to one significant figure.

## Conclusion

In summary, a ligand-based analytical method has been developed to identify and characterize multiple different binding sites present on the surfaces of αSyn aggregates. Using two-step competition binding assays to displace the fluorescent ligand ThT with four different ligands (BTA, OXI, S5H, and ThR), seven unique binding sites were identified across four different polymorphic preparations of αSyn fibrils (αSyn 1, αSyn 1s, αSyn 2, and αSyn 3). αSyn 1 has three different binding sites and does not bind the benzothiazole ligand BTA. αSyn 2 has four different binding sites and binds all of the ligands. αSyn 1s and αSyn 3 both have five binding sites, including one site that binds BTA but not ThT. Additionally, measurements of ligand dissociation constants reveal differences between similar binding sites on the four αSyn fibrils studied. For example, Site F on αSyn 3 has a relatively high affinity for ThT. The experiments also provide quantitative information about the populations of different ligand binding sites present on the fibrils.

These ligand binding assays allow characterization of different fibril morphologies and provide a detailed profile of the distribution of binding sites present. The approach represents a new, generally applicable ligand-based method that can be used to characterize and distinguish morphologically distinct fibrils composed of the same protein. There is scope to expand the range of ligands used to provide more detailed information on the binding site and to target a wide range of different types of protein aggregates in addition to αSyn. Future work will focus on applying this methodology to profile the binding sites present on amyloid fibrils formed in vivo.

## Supporting Information Available

Experimental details on the preparation and characterization of compounds and protein aggregates, of the binding assay methods used, and titration data including quantitative binding measurements (PDF)
