# Supplementary material for: Ligand Profiling to Characterize Different Polymorphic Forms of α-Synuclein Aggregates
Source: J Am Chem Soc. 2023 Nov 29;145(49):27030–7. doi: 10.1021/jacs.3c10521 (PMC10722502; doi:10.1021/jacs.3c10521)
Supplement: Supplementary file 1 — ja3c10521_si_001.pdf [file ja3c10521_si_001.pdf]

# Ligand profiling to characterise different polymorphic forms of α-synuclein aggregates

Timothy S. Chisholm [a] and Christopher A. Hunter [a,*]

[a] Yusuf Hamied Department of Chemistry, University of Cambridge, Lensfield Road, Cambridge CB2 1EW, UK. Email: herchelsmith.orgchem@ch.cam.ac.uk.

# Supporting Information

# Contents

Contents ........ 2

Materials and Instrumentation ........ 3

Chemical Synthesis ........ 4

Fluorescence Characterisation ........ 24

UV-Visible Characterisation ........ 24

Preparation of αSyn Fibrils ........ 24

Biophysical Characterisation of Amyloid Fibrils ........ 26

- Circular Dichroism Spectra ........ 26
- Transmission Electron Microscopy ........ 26

In Vitro Binding Assays ........ 27

- General Procedure for Fluorescence Titrations ........ 27
- Saturation Binding Assays ........ 27
- Fluorescence Anisotropy Binding Assays ........ 27
- One-Step Competition Binding Assays ........ 27
- One-Step Blocked Binding Assays ........ 27
- Two-Step Competition Binding Assays ........ 27
- Data fitting ........ 28

Photophysical Characterisation ........ 30

- UV-Visible Characterisation ........ 30
- Fluorescence Characterisation ........ 33

Dilution Series ........ 35

One-Step Blocked Binding Assays ........ 41

Tables of Quantitative Binding Measurements ........ 44

References ........ 49

## Materials and Instrumentation

All solvents and chemicals were obtained from commercial sources and used without further purification unless otherwise stated. Reactions were monitored by TLC or LCMS. TLC analyses were performed on Merck TLC Silica gel 60 $F_{254}$ glass plates (0.2 mm). LCMS analyses of samples were performed using a Waters Acquity H-class UPLC coupled with a single quadrupole Waters SQD2. An Acquity UPLC CSH C18 Column, 130Å, 1.7 µm, 2.1 mm x 50 mm was used as the UPLC column.

Purification of compounds by silica column chromatography were performed using an automated system (Combiflash® Rf+ or Combiflash® Rf+ Lumen) with prepackaged silica cartridges (25 µm or 50 µm PuriFlash® columns). $^{1}$H and $^{13}$C NMR spectra were recorded using a Bruker 600 MHz Avance 600 BBI spectrometer, a 500 MHZ Acance III Smart Probe spectrometer, or a 400 MHz Avance III HD Smart Probe spectrometer at 298.0 ± 0.1 K. Residual solvent peaks were used as an internal standard for calibration. All chemical shifts are quoted in ppm on the δ scale and the coupling constants are expressed in Hz. Signal splitting patterns are described as a singlet (s), broad singlet (br s), doublet (d), triplet (t), quartet (q), or multiplet (m).

HPLC-MS and HPLC-MS/MS analysis was performed on an Agilent 1100 Series LC system equipped with a G1310A isocratic pump, G1314A variable wavelength detector, G1316A thermostatted column compartment, and an Agilent 6300 Series Ion Trap. UV-vis spectra were collected on an Agilent Cary 60 UV-vis spectrophotometer controlled by Cary WinUV software. Fluorescence spectroscopic data were recorded using an Agilent Cary Eclipse Fluorescence Spectrophotometer controlled by Cary WinUV software, and equipped with a Cary Eclipse Automated Polarizer for anisotropy measurements. FT-IR spectra were collected with an ALPHA FT-IR Spectrometer from Bruker. Melting points were recorded with a Mettler Toledo MP90 melting point apparatus.

Protein LoBind (Eppendorf) microtubes were used for preparing and storing all solutions containing protein. Low retention pipette tips were used for all aqueous fluid handling.

## Chemical Synthesis

### 2-(4-(dimethylamino)phenyl)-3,6-dimethylbenzo[d]thiazol-3-ium chloride (Thioflavin T, ThT)

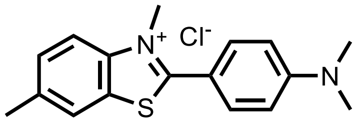

Thioflavin T (1.13 g, 3.54 mmol) was purchased from Sigma Aldrich with dye content ≥65%. Thioflavin T was recrystallised twice from hot water prior to use (0.53 g, 1.7 mmol, 47%).

***Aspect***: yellow crystalline solid. ***Yield***: 0.53 g (47%).

***$^{1}$H NMR (400 MHz, $CDCl_3$), δ(ppm):*** 8.05 – 7.97 (m, 2H), 7.86 (d, *J* = 8.5 Hz, 2H), 7.53 (d, *J* = 8.5 Hz, 1H), 6.82 (d, *J* = 8.4 Hz, 2H), 4.55 (s, 3H), 3.12 (s, 6H), 2.49 (s, 3H).

***$^{13}$C NMR (101 MHz, $CDCl_3$) δ(ppm):*** 172.57, 163.88, 154.15, 141.00, 139.09, 132.78, 131.27, 128.25, 123.44, 116.73, 112.40, 111.00, 77.48, 77.16, 76.84, 40.28, 39.65, 21.64.

***HRMS (ESI+):*** 283.1265 m/z: Calculated for $C_{17}H_{19}N_2S^+$ = 283.1269 $[M]^+$.

***IR (ATR, $cm^{-1}$):*** 3403, 1604, 1501, 1480, 1441, 1387, 1350, 1233, 1212, 1158, 827.

***MP:*** 195.5-196.5 °C

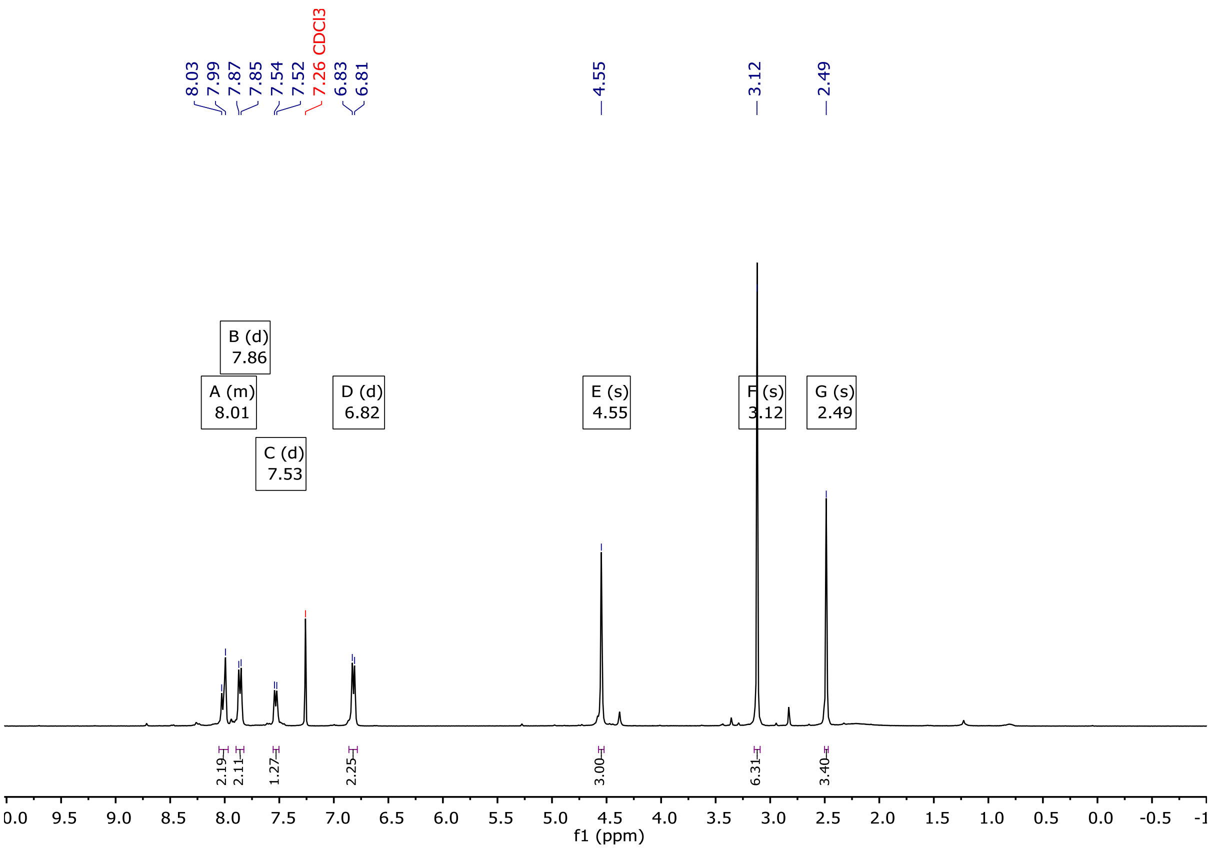


**Figure S1.** $^{1}H$ NMR (400 MHz, $CDCl_3$) spectra of ThT.

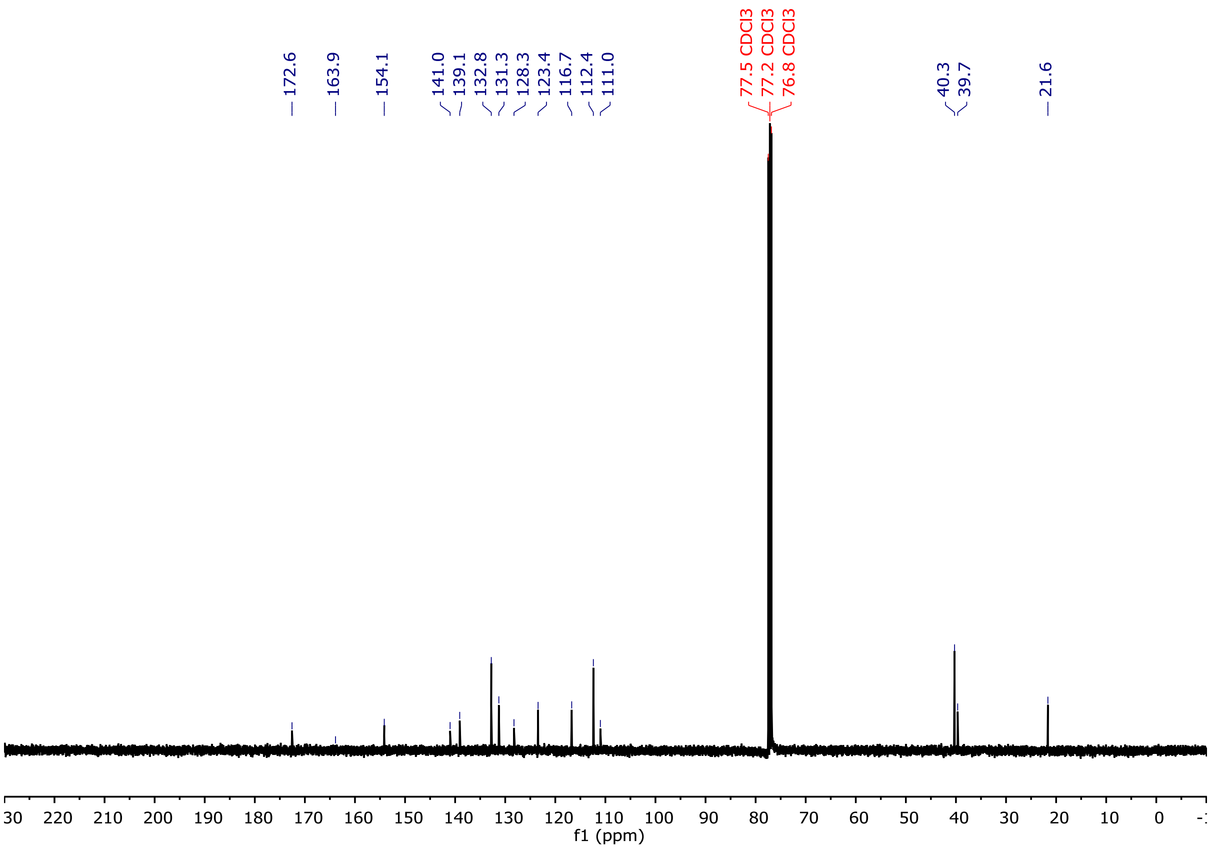


**Figure S2.** $^{13}C$ (101 MHz, $CDCl_3$) NMR spectra of ThT.

**(Z)-3-((E)-3-(4-nitrophenyl)allylidene)indolin-2-one (OXI)**

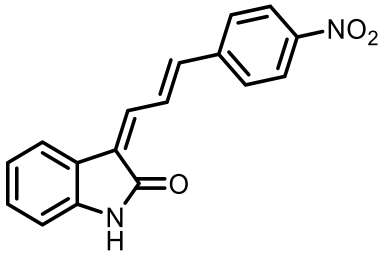


OXI was synthesised following a previously reported procedure.[1] A solution of 2-oxindole (3.61 g, 10 mmol) and 4-nitrocinnamaldehyde (1.77 g, 10 mmol, 1.0 equiv.) in acetic acid (25 mL, 2.5 mL/mmol) and 37% HCl (0.5 mL, 0.05 mL/mmol) was heated to reflux for 3 h. The resultant red solution was cooled to room temperature and diluted with distilled water (25 mL). The red precipitate was isolated by filtration and purified using silica column chromatography ($CH_2Cl_2$ to $CH_2Cl_2$:EtOAc 95:5) to afford OXI as a red crystalline solid (1.36 g, 4.65 mmol, 47%).

***Aspect***: red solid. ***Yield***: 1.36 g (47%).

***$^1$H NMR (400 MHz, $CDCl_3$), δ(ppm):*** 10.60 (s, 1H), 8.63 (dd, *J* = 15.8, 11.5 Hz, 1H), 8.27 (d, *J* = 8.8 Hz, 2H), 7.82 (d, *J* = 8.8 Hz, 2H), 7.61 (s, 1H), 7.59 (d, *J* = 5.2 Hz, 1H), 7.28 (d, *J* = 15.7 Hz, 1H), 7.22 (t, *J* = 7.2 Hz, 1H), 6.97 (t, *J* = 7.6 Hz, 1H), 6.82 (d, *J* = 7.7 Hz, 1H).

***$^{13}$C NMR (101 MHz, $CDCl_3$) δ(ppm):*** 168.13, 147.04, 142.70, 139.09, 134.14, 129.71, 128.16, 127.56, 124.29, 123.31, 121.29, 120.46, 109.67, 40.15, 39.94, 39.73, 39.52, 39.31, 39.10, 38.89.

***HRMS (ESI+):*** 293.0921 m/z: Calculated for $C_{17}H_{13}N_2O_3^+$ = 293.0926 $[M+H]^+$.

***IR (ATR, $cm^{-1}$):*** 2924, 2849, 2361, 2334, 1691, 1603, 1585, 1548, 1510, 1468, 1339, 1214, 1180, 1109, 978, 869, 838.

***Decomposition point:*** decolorisation at 254 °C, melting at 260.2-261.2 °C with effervescence.

Characterisation data is in agreement with that reported by Chu *et al.*[1]

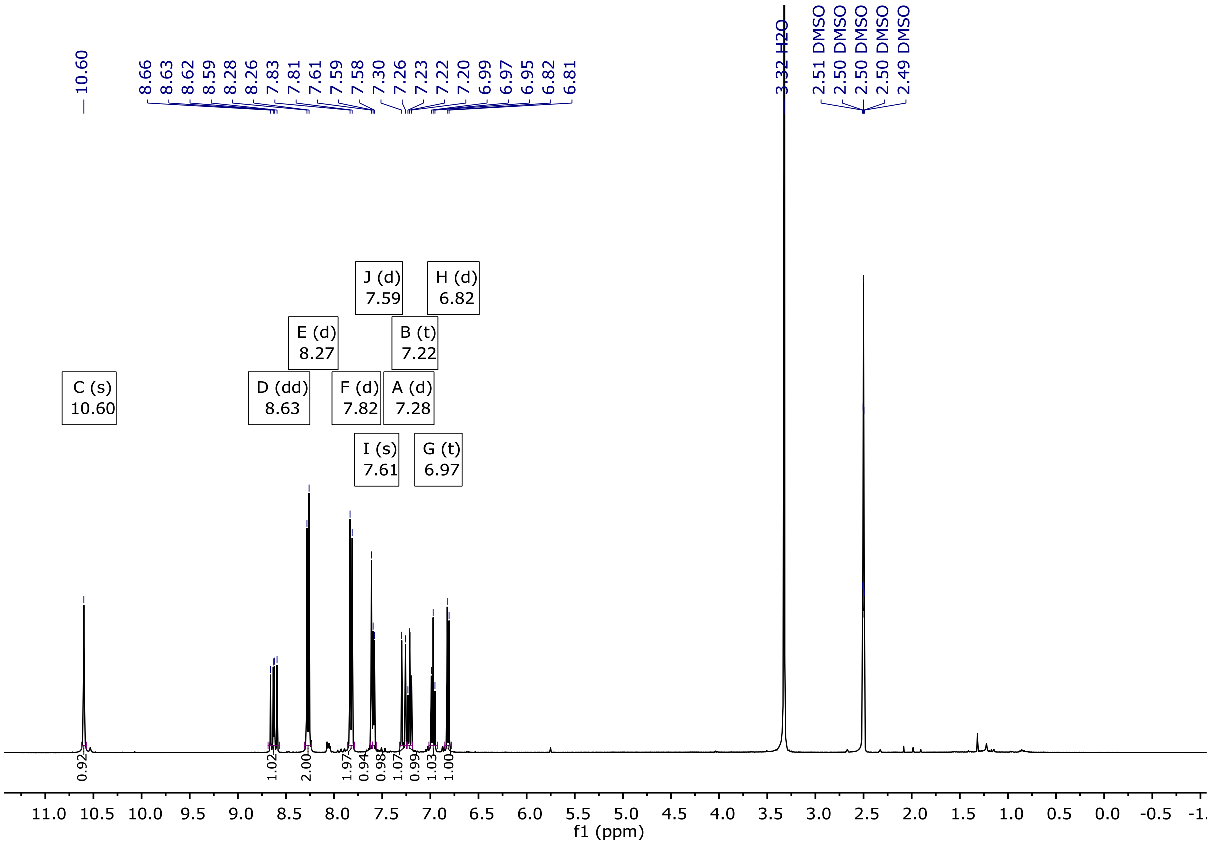


**Figure S3**. $^{1}H$ NMR (400 MHz, $CDCl_3$) spectra of OXI.

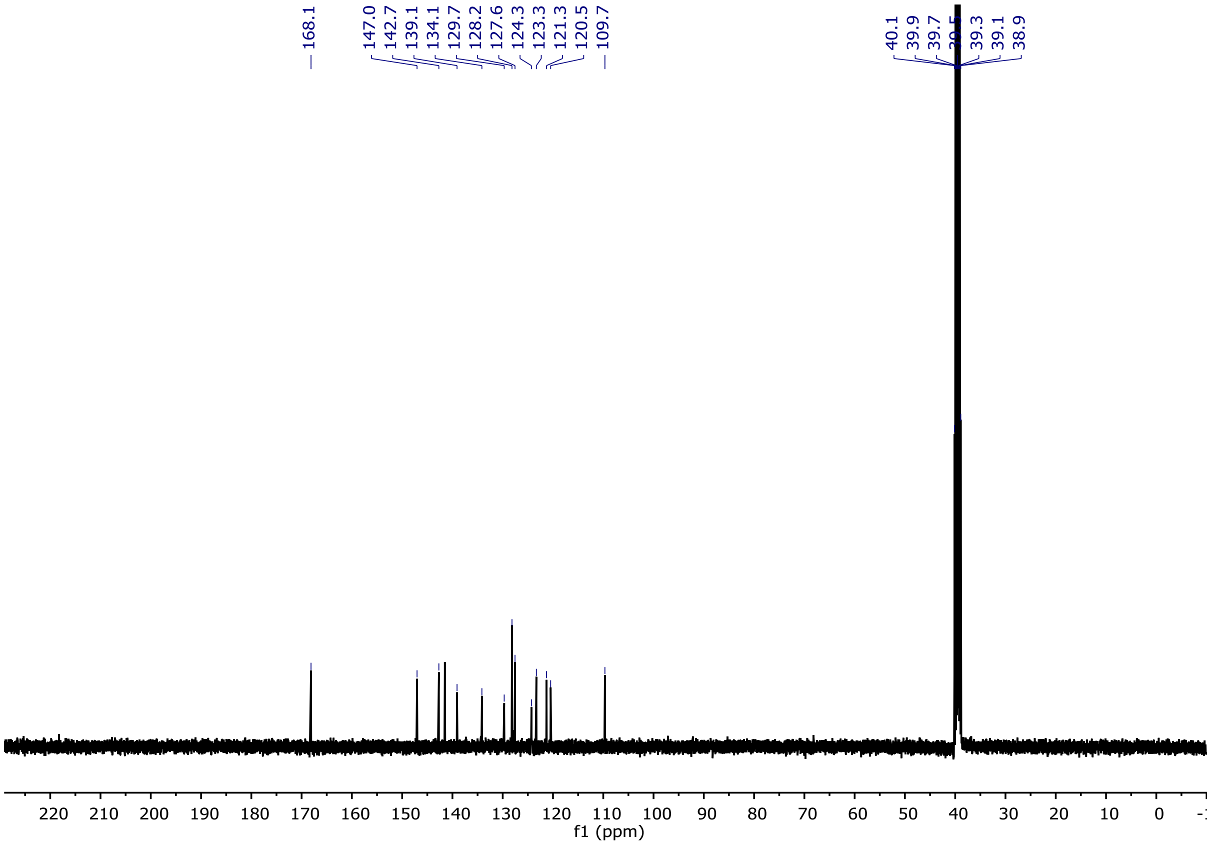


**Figure S4**. $^{13}C$ NMR (101 MHz, $CDCl_3$) spectra of OXI.

**(*E*)-3-(4-(benzyloxy)phenyl)acrylic acid (S1)**

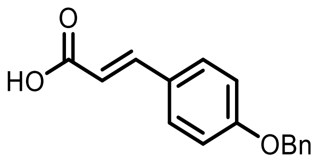

To coumaric acid (1.31 g, 7.98 mmol) in absolute ethanol (8.0 mL) was added benzyl bromide (1.96 mL, 16.5 mmol) then sodium hydroxide (40 g, 39.8 mmol) in distilled water (8.0 mL). The resultant yellow suspension was stirred for 5 min until clear, then refluxed for 36 h. The reaction was then cooled in an ice bath and poured into ice cold water (50 mL) and quenched with 5 M HCl. The resultant precipitate was isolated by filtration, washed with ice cold water (3 x 10 mL) then dried *in vacuo* to afford (*E*)-3-(4-(benzyloxy)phenyl)acrylic acid **S1** as a white solid (2.03 g, 7.98 mmol, 100%).

***Aspect***: yellow solid. ***Yield***: 2.03 g (100%).

***$^{1}$H NMR (400 MHz, $CDCl_3$), δ(ppm):*** 7.57 (d, J = 8.3 Hz, 2H), 7.48 – 7.31 (m, 6H), 7.02 (d, J = 8.2 Hz, 2H), 6.36 (d, J = 16.0 Hz, 1H), 5.15 (s, 2H).

***HRMS (ESI+):*** 255.1010 m/z: Calculated for $C_{16}H_{15}O_3^+$ = 255.1016 $[M+H]^+$.

***IR (ATR, cm$^{-1}$):*** 2911, 1669, 1601, 1544, 1510, 1427, 1413, 1304, 1289, 1241, 1172, 1014, 981, 968, 920, 825.

***MP:*** 205.9-206.9 °C

Characterisation data is in agreement with that reported by Lee *et al.*[2]

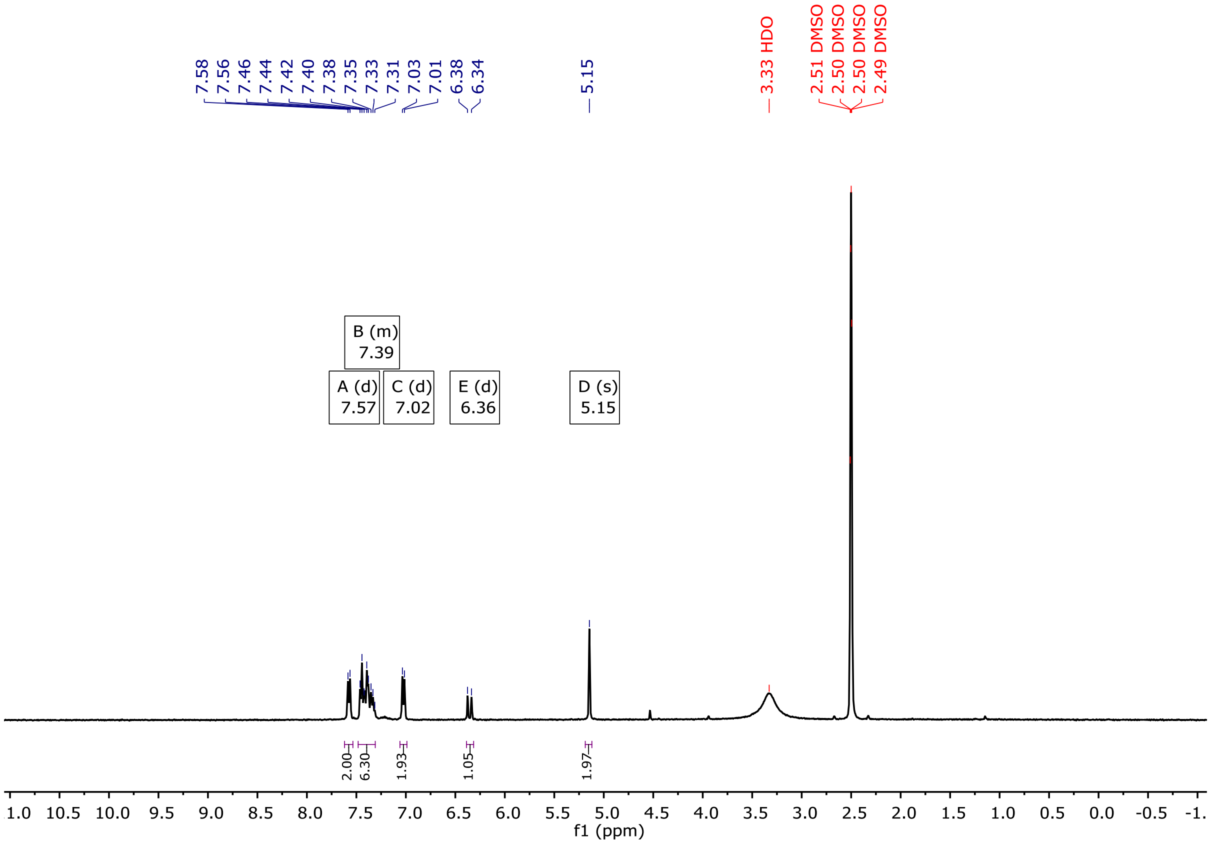


**Figure S5**. $^{1}$H NMR (400 MHz, $CDCl_3$) spectra of **S1**.

**(E)-3-(4-(benzyloxy)phenyl)acryloyl chloride (S2)**

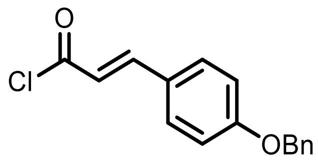


**S1** (2.03 g, 7.98 mmol) was solvated in oxalyl chloride (70 mL, 8.8 mL/mmol) at 0 °C, then warmed to room temperature and stirred for 2 h. The excess oxalyl chloride was removed *in vacuo* to afford **S2** as a white solid (2.02 g, 7.41 mmol, 93%).

***Aspect***: yellow solid. ***Yield***: 2.02 g (93%).

***$^{1}$H NMR (400 MHz, CDCl$_3$), δ(ppm):*** 7.79 (d, *J* = 15.4 Hz, 1H), 7.54 (d, *J* = 8.4 Hz, 2H), 7.45 – 7.34 (m, 5H), 7.02 (d, *J* = 8.4 Hz, 2H), 6.51 (d, *J* = 15.5 Hz, 1H), 5.13 (s, 2H).

***$^{13}$C NMR (101 MHz, CDCl$_3$) δ(ppm):*** 166.3, 162.1, 150.6, 146.7, 136.2, 131.3, 130.2, 128.9, 128.8, 128.5, 128.5, 127.6, 126.2, 119.9, 115.7, 77.5, 77.2, 76.8, 70.4.

***HRMS (ESI+):*** 273.0674 m/z: Calculated for $C_{16}H_{14}ClO_2^+$ = 273.0677 $[M+H]^+$.

Characterisation data is in agreement with that reported by Lee *et al.*[2]

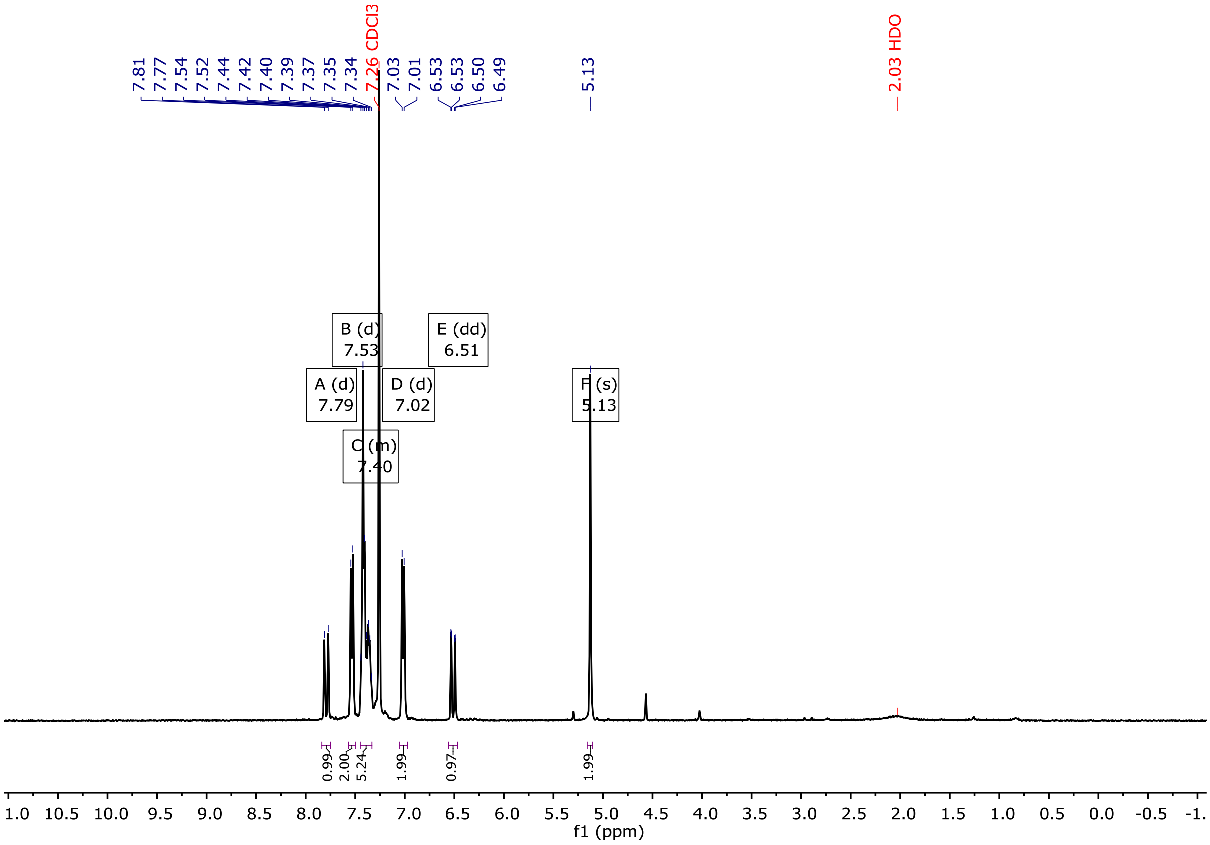


**Figure S6.** $^{1}H$ NMR (400 MHz, $CDCl_3$) spectra of **S2**.

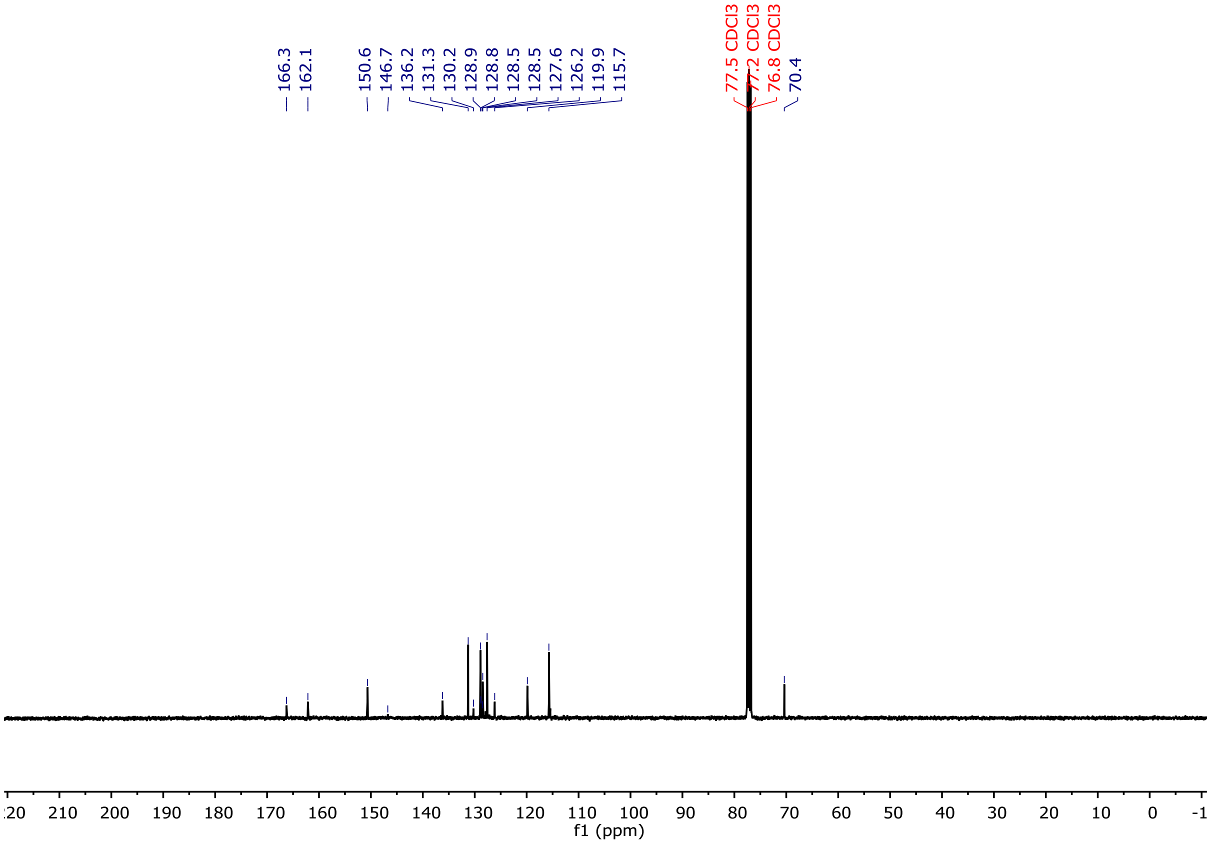


**Figure S7.** $^{13}C$ NMR (101 MHz, $CDCl_3$) spectra of **S2**.

**(2Z,4E)-5-(4-(benzyloxy)phenyl)-3-hydroxy-1-(thiazol-2-yl)penta-2,4-dien-1-one (S3)**

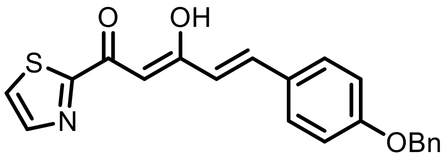


To a solution of 1-(thiazol-2-yl)ethan-1-one (340 µL, 3.3 mmol, 1.0 equiv.) in THF (10 mL) at -78 °C was added 0.94 M lithium bis(trimethylsilyl)amide in THF (LiHMDS, 3.9 mL, 3.63 mmol, 1.1 eq.) dropwise. The resultant solution was stirred at -78 °C for 1 h. To the reaction mixture was then added **S2** (990 mg, 3.63 mmol, 1.2 equiv.) in THF (10 mL) dropwise, and the reaction was stirred for a further 3 h at -78 °C. The reaction mixture was then warmed to room temperature and diluted with saturated $NH_4Cl$ (20 mL) then extracted with EtOAc (2 x 20 mL). The combined organic extracts were washed with distilled $H_2O$ (20 mL), dried over anhydrous sodium sulfate, and concentrated *in vacuo.* The crude product was purified using silica column chromatography (PE to PE:EtOAc 3:1) to afford **S3** as a yellow solid (350 mg, 0.96 mmol, 30%).

***Aspect***: yellow solid. ***Yield***: 0.35 g (30%).

***$^1$H NMR (400 MHz, $CDCl_3$), δ(ppm):*** 15.03 (s, 1H), 8.00 (d, *J* = 3.1 Hz, 1H), 7.66 – 7.60 (m, 2H), 7.50 (d, *J* = 8.7 Hz, 2H), 7.40 (td, *J* = 16.3, 15.1, 7.4 Hz, 7H), 6.99 (d, *J* = 8.7 Hz, 2H), 6.74 (s, 1H), 6.51 (d, *J* = 15.8 Hz, 1H), 5.09 (s, 2H).

***$^{13}$C NMR (101 MHz, $CDCl_3$) δ(ppm):*** 182.4, 178.2, 167.3, 160.7, 144.8, 140.6, 136.4, 132.5, 129.9, 128.7, 128.2, 127.8, 127.8, 127.5, 127.5, 125.3, 120.0, 115.4, 114.3, 96.9, 77.4, 77.1, 76.8, 70.1.

***HRMS (ESI+):*** 361.1023 m/z: Calculated for $C_{17}H_{13}N_2OS^+$ = 293.0926 [M+H]$^+$.

***IR (ATR, cm$^{-1}$):*** 3110, 3086, 3065, 3033, 2923, 2853, 1628, 1562, 1509, 1481, 1437, 1422, 1383, 1330, 1311, 1283, 1244, 1164, 1114, 1079, 1060, 1036, 1026, 972, 958, 872, 825.

***MP:*** 112.3-117.8 °C

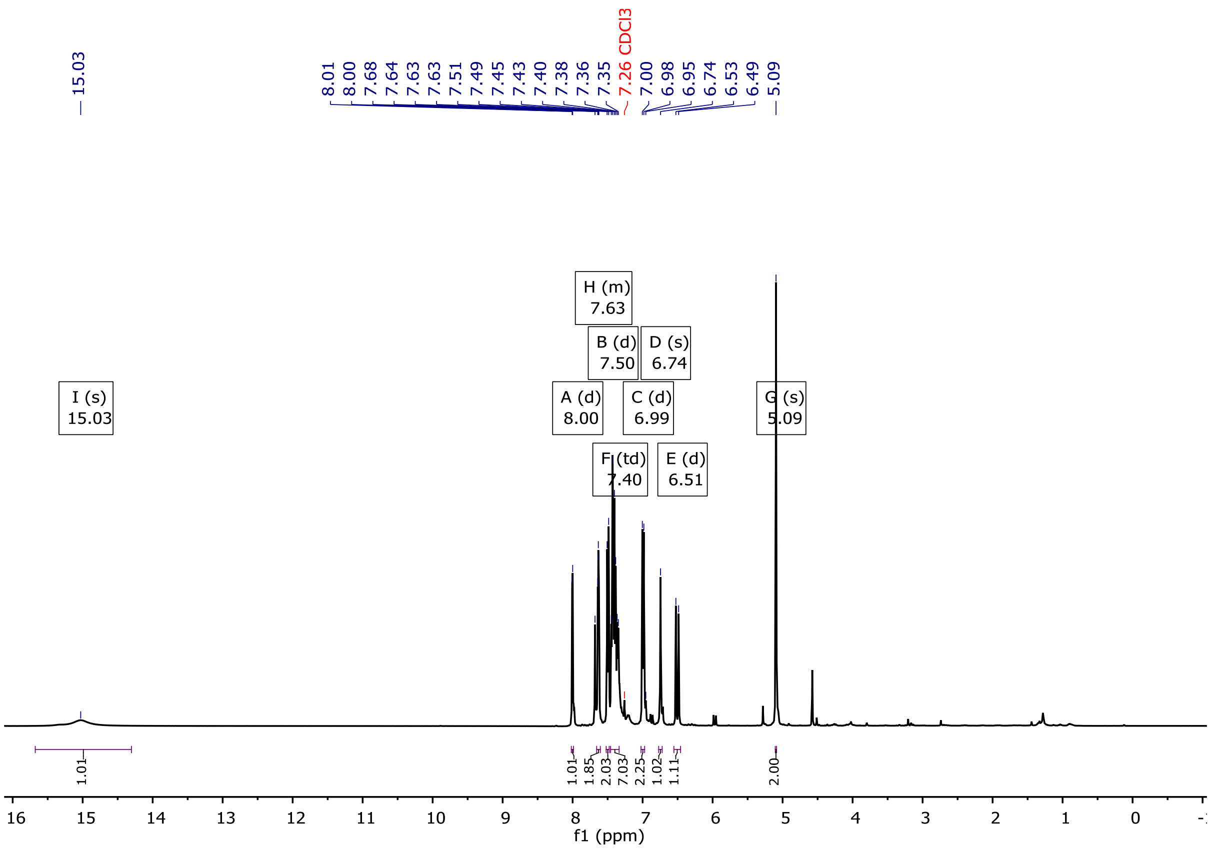


**Figure S8.** $^{1}H$ NMR (400 MHz, $CDCl_3$) spectra of **S3**.

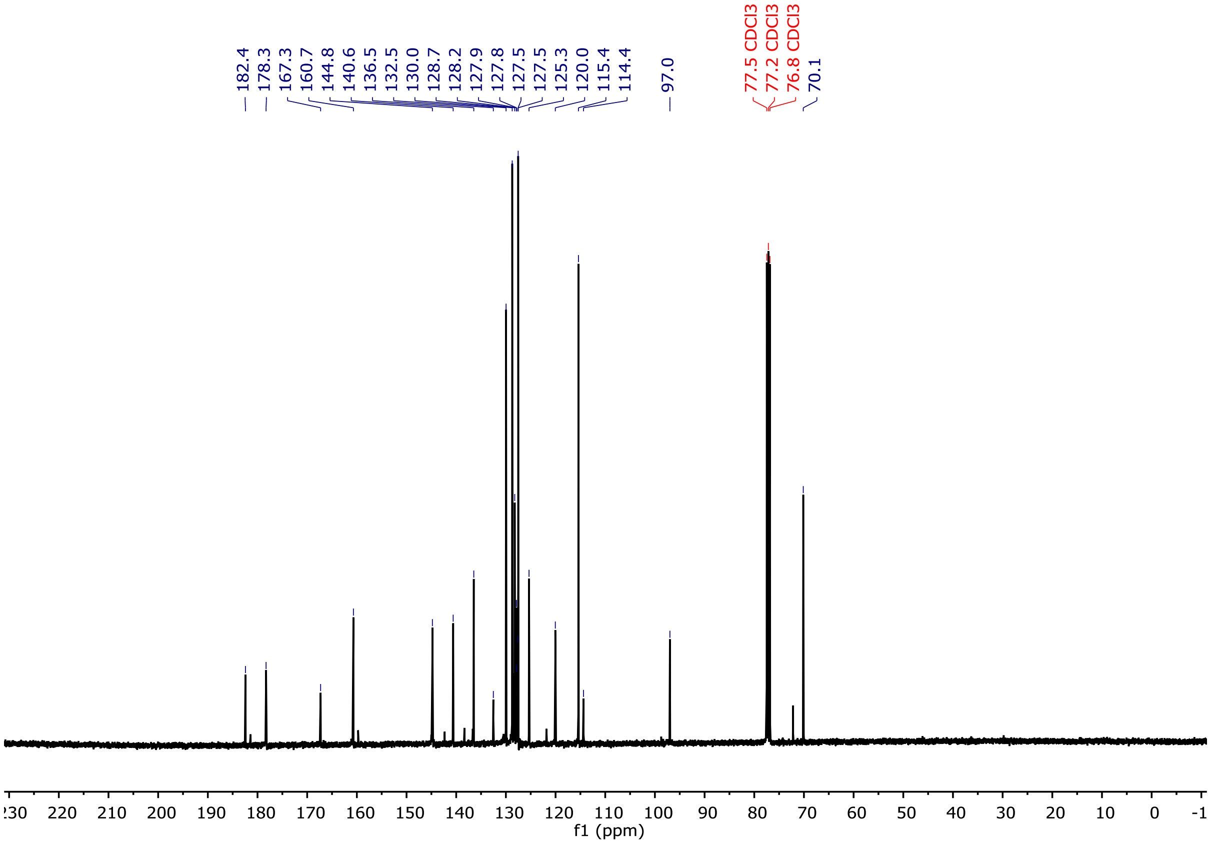


**Figure S9.** $^{13}C$ NMR (101 MHz, $CDCl_3$) spectra of **S3**.

## (E)-5-(4-(benzyloxy)styryl)-3-(thiazol-2-yl)isoxazole (S5H)

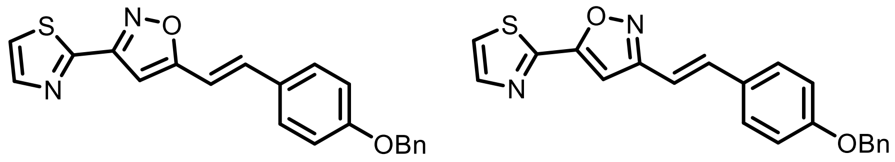


S5H was synthesised based on a previously reported procedure.[3] To a suspension of **S3** (255 mg, 0.700 mmol) in EtOH (4 mL, 5.7 mmol/mL) was added $NH_2OH{\cdot}HCl$ (116 mg, 196 µL, 2.80 mmol, 4.0 equiv.). The mixture was stirred at 80 °C for 6 h, then cooled and the solvent removed *in vacuo.* The crude product was purified using silica column chromatography (PE to PE:EtOAc 3:1) to afford an approximately 1:1 mixture of two isomers S5H as a crystalline yellow solid (181 mg, 0.502 mmol, 72%). This mixture was used without further purification as previously reported.[3]

***Aspect***: yellow oil. ***Yield***: 181 mg (72%).

***$^1$H NMR (400 MHz, CDCl$_3$), δ(ppm):*** 7.98 (dd, J = 12.6, 3.2 Hz, 2H), 7.56 – 7.30 (m, 17H), 7.20 (d, J = 16.4 Hz, 1H), 7.03 (dd, J = 26.8, 8.6 Hz, 6H), 6.88 (d, J = 16.4 Hz, 1H), 6.80 (s, 1H), 5.11 (s, 4H).

***$^{13}$C NMR (101 MHz, CDCl$_3$), δ(ppm)*** 170.2, 163.8, 162.9, 160.0, 159.8, 158.8, 156.9, 154.5, 144.6, 143.8, 136.7, 136.7, 136.5, 135.4, 128.9, 128.8, 128.7, 128.4, 128.3, 128.2, 127.6, 121.5, 121.0, 115.4, 115.4, 113.3, 110.7, 99.0, 98.9, 77.5, 77.2, 76.8, 70.2.

***HRMS (ESI+):*** 361.1023 m/z: Calculated for $C_{21}H_{17}N_2O_2S^+$ = 361.1011 $[M+H]^+$.

***IR (ATR, cm$^{-1}$):*** 3107, 2921, 2853, 1643, 1603, 1579, 1514, 1454, 1430, 1383, 1316, 1301, 1254, 1173, 1157, 1140, 1110, 1079, 1060, 1037, 1028, 987, 962, 917, 872, 818.

***MP:*** 142.2-143.7 °C

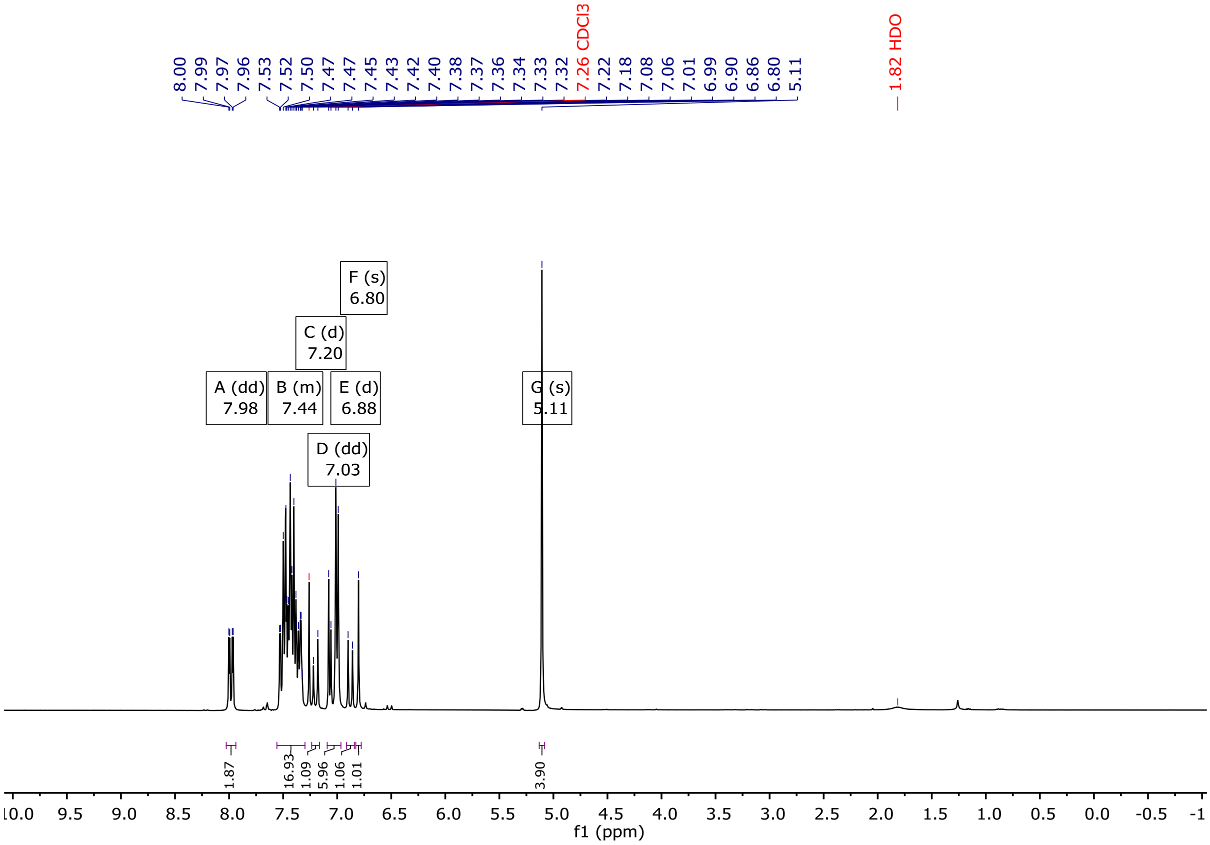


**Figure S10**. $^{1}$H NMR (400 MHz, $CDCl_3$) spectra of S5H.

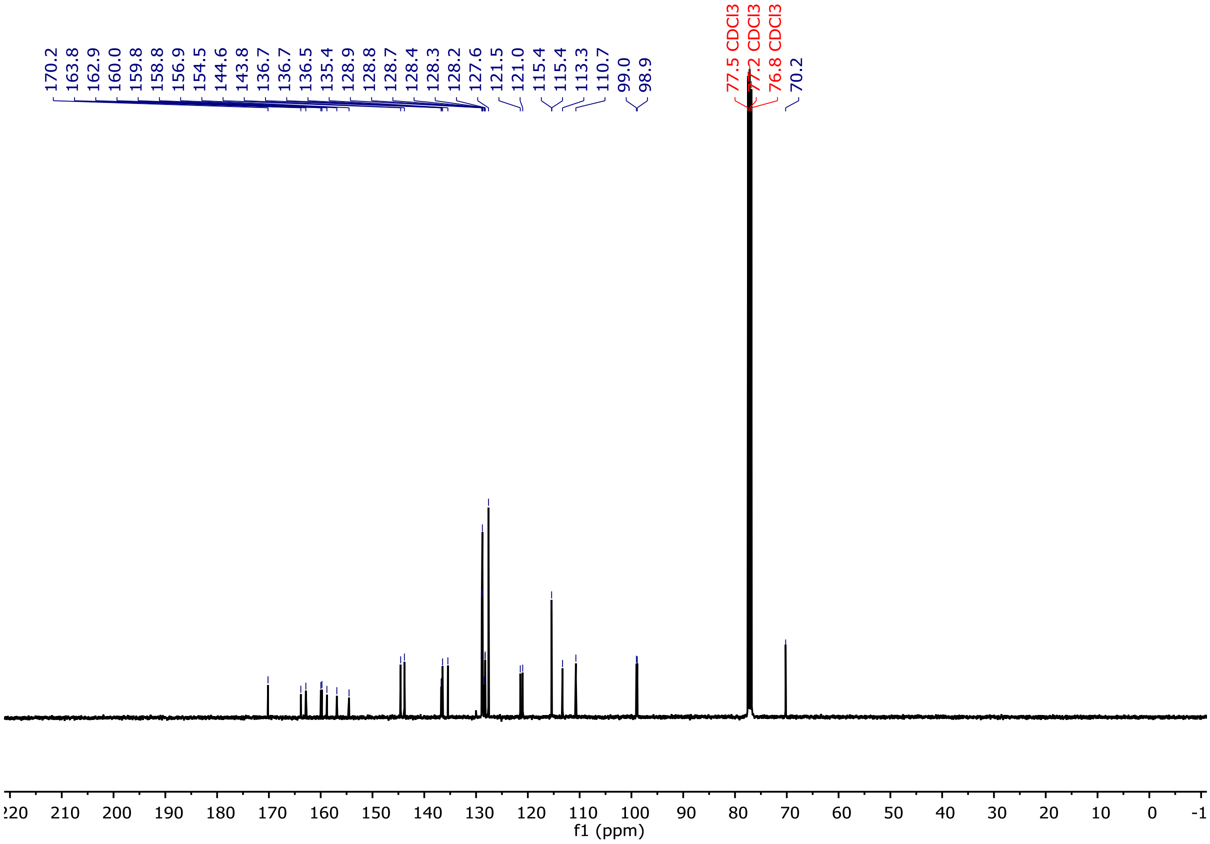


**Figure S11.** $^{13}$C NMR (101 MHz, $CDCl_3$) spectra of S5H.

**2-amino-5-methoxybenzenethiol (S4)**

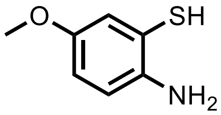


**S4** was synthesised following a previously reported procedure.[4] To a suspension of 2-amino-6-methoxybenzothiazole (20.2 g, 112 mmol) and potassium hydroxide (62.3 g, 1.11 mol) in degassed water (250 mL) was added ethyleneglycol (27.9 mL). This clear solution was refluxed for 21 h, and the resultant dark green solution was cooled to room temperature and neutralised with acetic acid. The formed precipitate was collected using vacuum filtration and washed with cold water (200 mL) then dried *in vacuo* to afford **S4** as a brown solid (17.4 g, 112 mmol, 100%).

***Aspect***: brown solid. ***Yield***: 17.4 g (100%).

***$^{1}$H NMR (400 MHz, DMSO-$d^6$), δ(ppm):*** 6.83 (dd, *J* = 8.7, 3.0 Hz, 1H), 6.71 (d, *J* = 8.7 Hz, 2H), 3.63 (s, 3H).

***$^{13}$C NMR (101 MHz, DMSO-$d^6$), δ(ppm):*** 152.0, 142.7, 120.2, 119.5, 119.4, 116.8, 55.90.

***HRMS (ESI+):*** 155.0407, 309.0718 m/z: Calculated for $C_{14}H_{18}N_2O_2S_2^{2+}$ = 155.0400 [disulfide+2H]$^{2+}$, $C_{14}H_{17}N_2O_2S_2^{+}$ = 309.0726 [disulfide+H]$^{+}$

***FT-IR (ATR)***: 3444, 3351, 2927, 2831, 1595, 1490, 1268, 1035, 816.

***Decomposition point:*** decolorisation at 57 °C, turned black at 75 °C, melted at 78.0-79.0 °C.

Characterisation data is in agreement with that reported by Qin *et al.*[4]

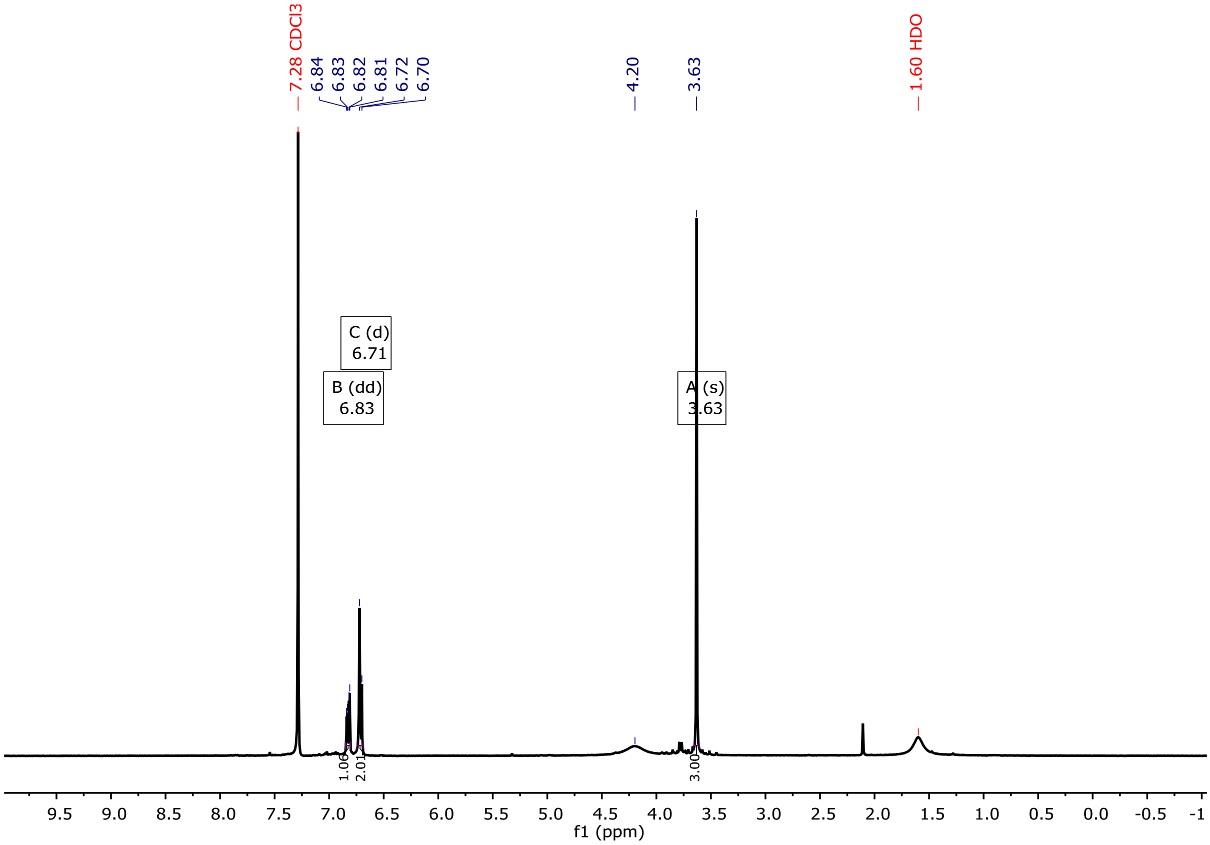


**Figure S12.** $^{1}H$ NMR (400 MHz, DMSO-$d^{6}$) spectra of **S4**.

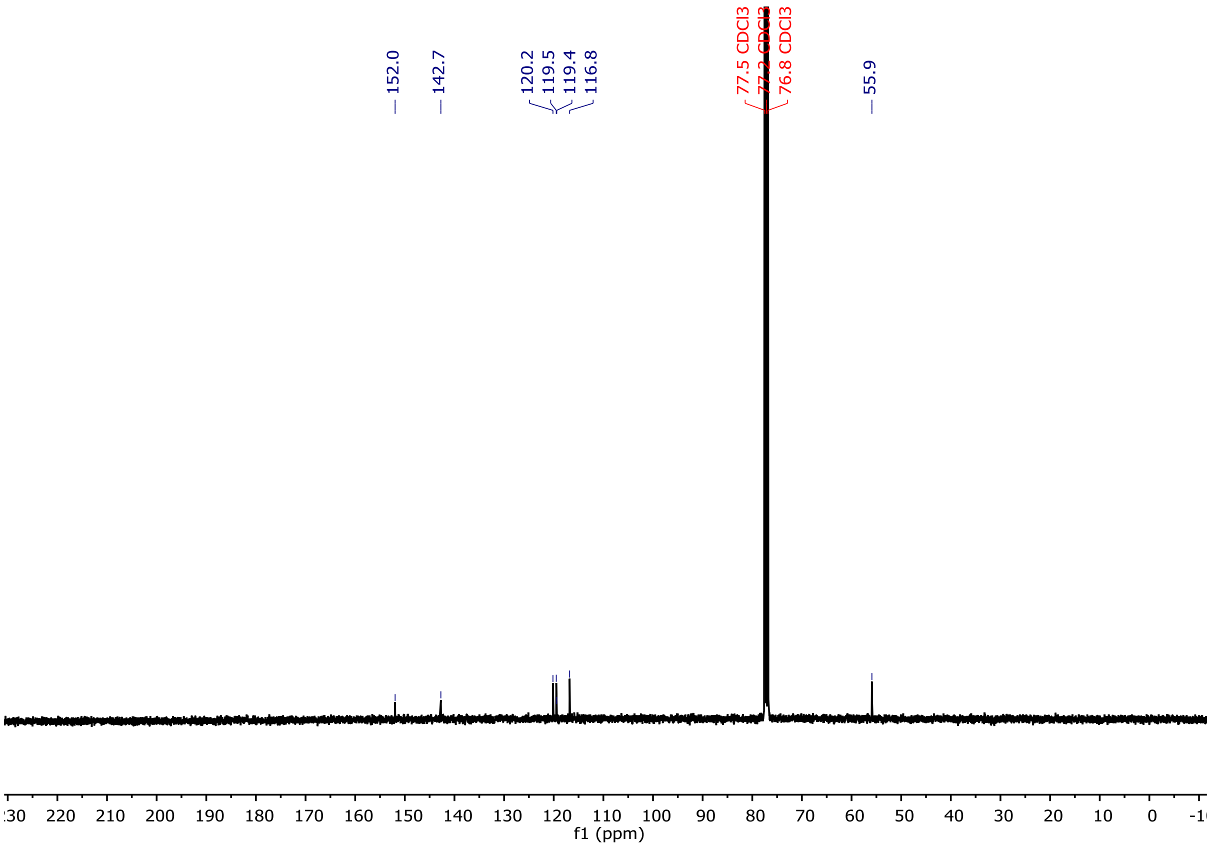


**Figure S13.** $^{13}C$ NMR (101 MHz, DMSO-$d^{6}$) spectra of **S4**.

**4-(6-methoxybenzo[*d*]thiazol-2-yl)-N,N-dimethylaniline (S5)**

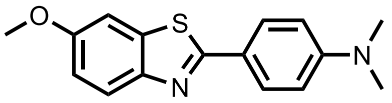

**S5** was synthesised following a previously reported procedure.[4] To a solution of **S4** (17.3 g, 111 mmol) in DMSO (200 mL) was added 4-(dimethylamino)-benzaldehyde (82.8 g, 555 mmol) and the dark green solution stirred at 180 °C for 20 min. The reaction mixture was cooled to room temperature and a mixture of ethyl acetate:water (1:1, 200 mL) was added. The resultant precipitate was collected by vacuum filtration and washed with water (350 mL) then ethanol (450 mL) then dried *in vacuo* to afford **S5** as a yellow solid (20.8 g, 73.3 mmol, 64%).

***Aspect***: yellow solid. ***Yield***: 20.8 g (64%).

***$^{1}$H NMR (400 MHz, DMSO-$d^6$), δ(ppm):*** 7.82 (t, *J* = 7.9 Hz, 3H), 7.62 (s, 1H), 7.06 (d, *J* = 8.6 Hz, 1H), 6.81 (d, *J* = 8.1 Hz, 2H), 3.83 (s, 3H), 3.01 (s, 6H).

***$^{13}$C NMR (101 MHz, DMSO-$d^6$), δ(ppm):*** 165.8, 157.3, 152.4, 148.8, 135.7, 128.5, 122.8, 120.9, 116.2, 115.63, 112.3, 105.4, 56.2.

***HRMS (ESI+):*** 285.1057 m/z: Calculated for $C_{16}H_{17}N_2OS^+$ = 285.1062 $[M+H]^+$.

***IR (ATR)***: 1606, 1462, 1431, 1368, 1222, 1166, 1058, 1023, 946, 832.

***Decomposition point:*** decolorisation at 234.5 °C, melted at 242.3-243.3 °C, generated a dark vapour at 243.4 °C.

Characterisation data is in agreement with that reported by Qin *et al.*[4]

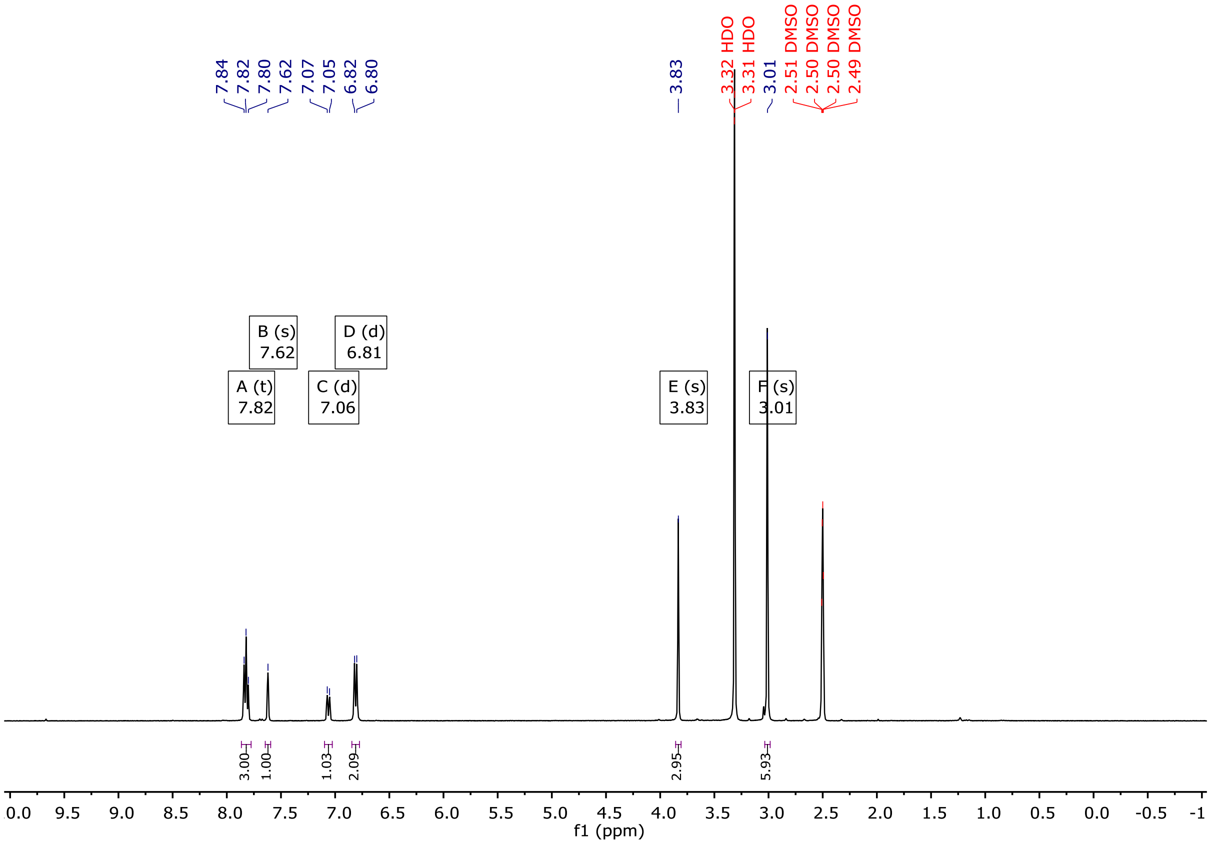


**Figure S14.** $^{1}H$ NMR (400 MHz, DMSO-$d^6$) spectra of **S5**.

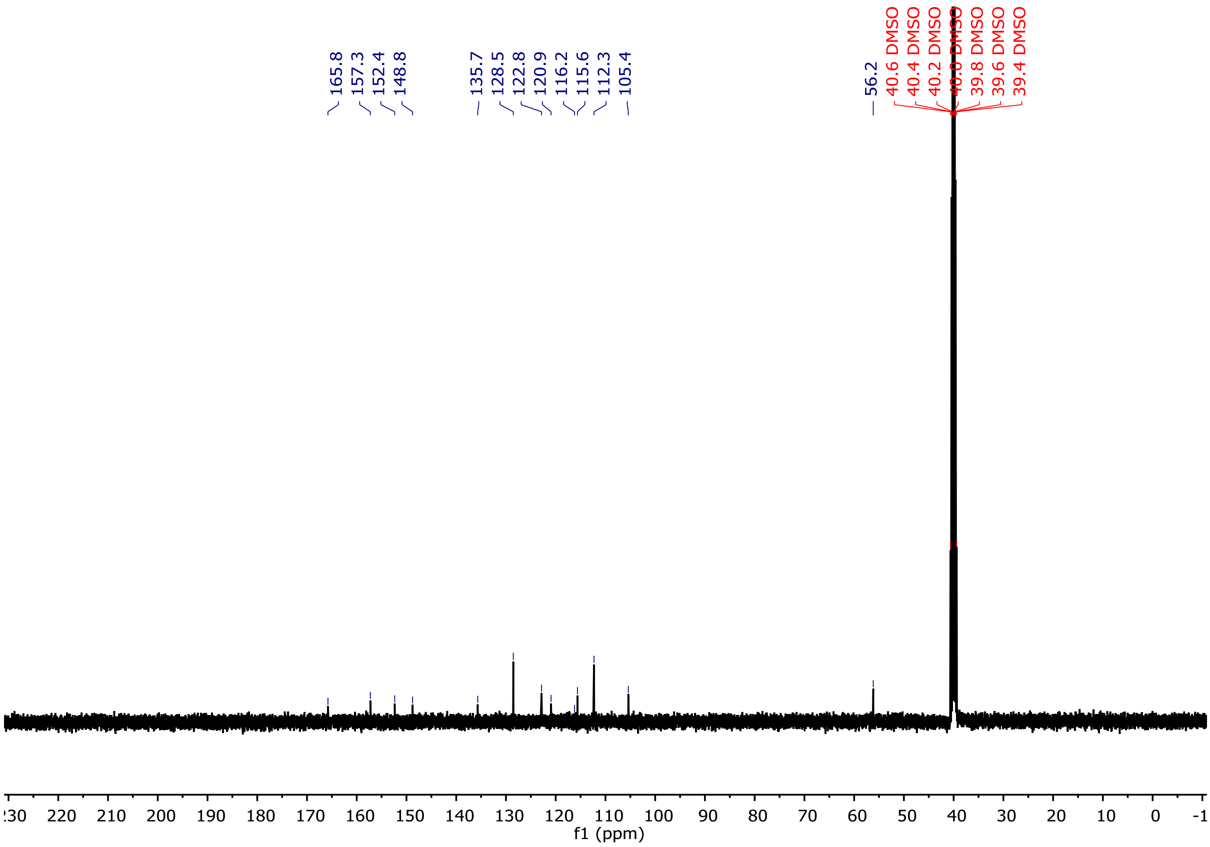


**Figure S15.** $^{13}C$ NMR (101 MHz, DMSO-$d^6$) spectra of **S5**.

**2-(4-(dimethylamino)phenyl)benzo[*d*]thiazol-6-ol (S6)**

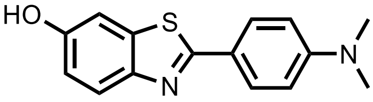

**S6** was synthesised following a previously reported procedure.[4] To a solution of **S5** (21.0 g, 73.8 mmol) in anhydrous dichloromethane (800 mL) was added boron tribromide (242 mL, 1 M in dichloromethane) dropwise at 0 °C. The reaction mixture was warmed to room temperature and stirred for 17 h, then quenched by the addition of water (200 mL) then saturated sodium bicarbonate solution (200 mL). The precipitate was collected by vacuum filtration and washed with water (200 mL) then dichloromethane (200 mL) and dried *in vacuo* to afford **S6** as an orange solid (19.7 g, 72.9 mmol, 99%).

***Aspect***: orange solid. ***Yield***: 19.7 g (99%).

***$^1$H NMR (400 MHz, DMSO-$d^6$), δ(ppm):*** 7.80 (d, *J* = 8.9 Hz, 2H), 7.72 (d, *J* = 8.7 Hz, 1H), 7.33 (d, *J* = 2.4 Hz, 1H), 6.92 (dd, *J* = 8.8, 2.4 Hz, 1H), 6.81 (d, *J* = 9.0 Hz, 2H), 3.01 (s, 6H).

***$^{13}$C NMR (101 MHz, DMSO), δ(ppm):*** 164.4, 155.2, 151.5, 146.2, 134.8, 128.1, 122.1, 115.8, 112.6, 106.9.

***HRMS (ESI+):*** 271.0905 m/z: Calculated for $C_{15}H_{15}N_2OS^+$ = 271.0900 [M+H]$^+$.

***IR (ATR)***: 2956, 2920, 2851, 1606, 1475, 1427, 1371, 1339, 1278, 1195, 810.

***Decomposition point***: decolorisation at 230 °C, turned black at 240 °C, melted at 241.9-243.1 °C.

Characterisation data is in agreement with that reported by Qin *et al.*[4]

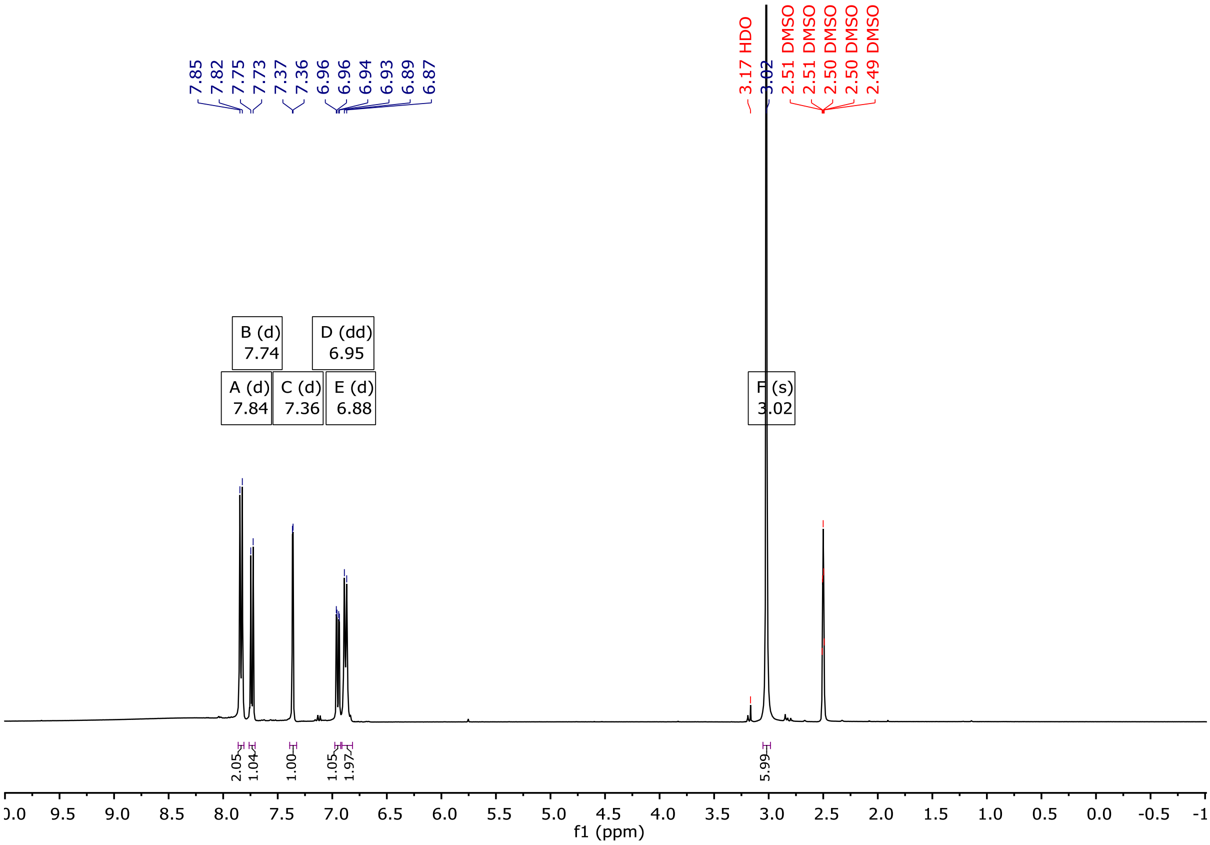


**Figure S16.** $^{1}H$ NMR (400 MHz, DMSO-$d^6$) spectra of **S6**.

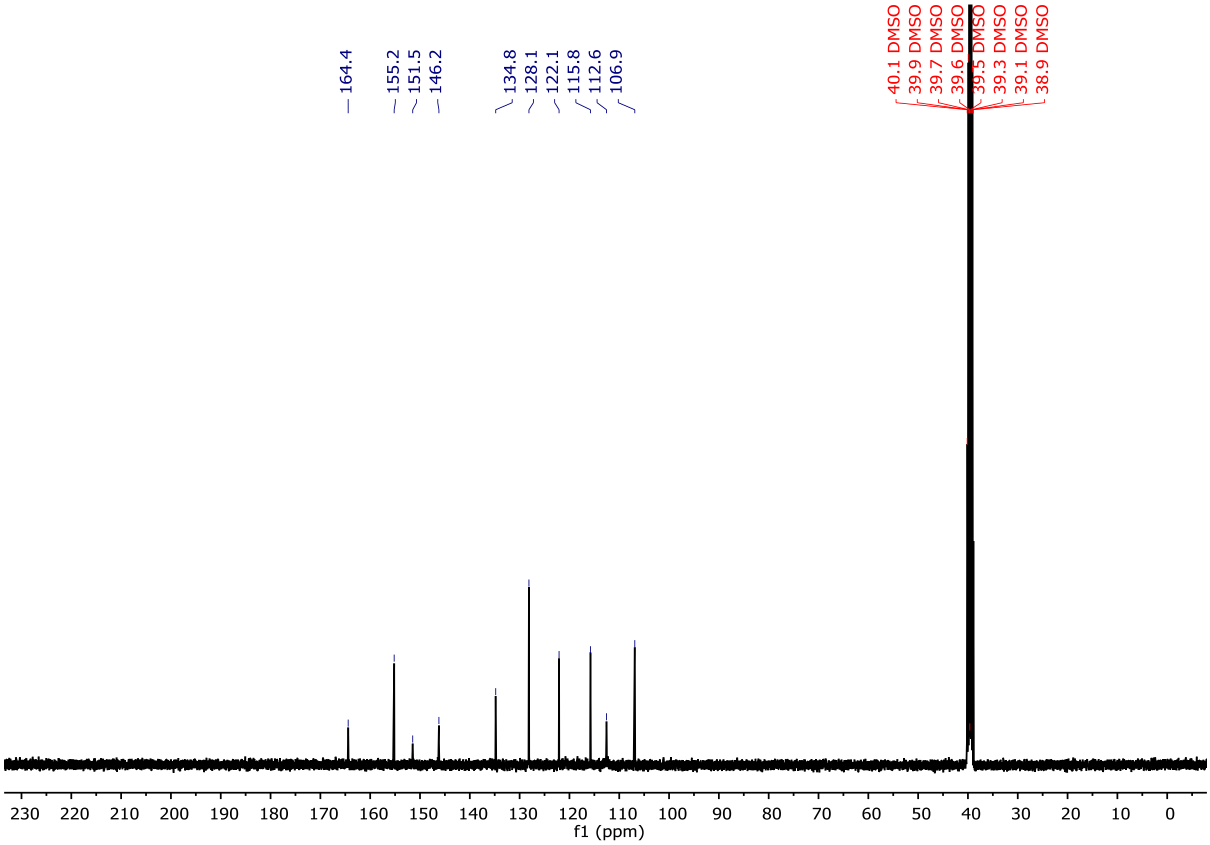


**Figure S17.** $^{13}C$ NMR (101 MHz, DMSO-$d^6$) spectra of **S6**.

**2-(2-(2-((2-(4-(dimethylamino)phenyl)benzo[*d*]thiazol-6-yl)oxy)ethoxy)ethoxy)ethan-1-ol (BTA)**

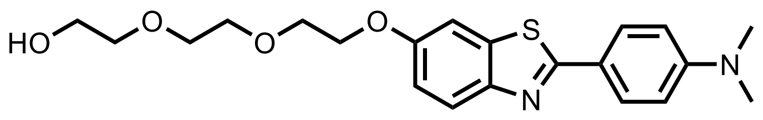


To a suspension of **S6** (503 mg, 1.86 mmol) and triphenylphosphine (1.21 g, 4.61 mmol) in anhydrous THF (100 mL) was added triethylene glycol (618 µL, 4.63 mmol) then DIAD (911 µL, 4.63 mmol). The reaction mixture was stirred at room temperature for 23 h. The solvent was evaporated under reduced pressure and the residue purified by flash chromatography (EtOAc:MeOH, 10:0 to 9:1) to afford BTA as a white solid (495 mg, 1.23 mmol, 67%).

***Aspect***: white solid. ***Yield***: 495 mg (67%).

***$^{1}$H NMR (400 MHz, CDCl$_3$) δ*** 7.86 (d, *J* = 8.6 Hz, 2H), 7.83 (d, *J* = 8.9 Hz, 1H), 7.29 (d, *J* = 2.5 Hz, 1H), 7.03 (dd, *J* = 8.9, 2.5 Hz, 1H), 6.68 (d, *J* = 8.6 Hz, 2H), 4.13 (t, *J* = 4.8 Hz, 2H), 3.83 (t, *J* = 4.7 Hz, 2H), 3.73 – 3.68 (m, 4H), 3.68 – 3.64 (m, 2H), 3.58 (t, *J* = 4.6 Hz, 2H), 2.98 (s, 6H).

***$^{13}$C NMR (101 MHz, CDCl$_3$)*** 166.8, 156.3, 152.1, 149.3, 135.9, 128.7, 122.9, 121.7, 115.6, 111.9, 105.6, 77.5, 77.2, 76.8, 72.6, 71.0, 70.6, 69.9, 68.2, 61.9, 40.3.

***HRMS (ESI+):*** m/z: Calculated for $C_{21}H_{27}N_2O_4S^+$ = 403.1686 [M+H]$^+$.

***IR (ATR, cm$^{-1}$):*** 2922, 2884, 1606, 1559, 1492, 1449, 1365, 1349, 1285, 1261,1223, 1188, 1138, 1126, 1101, 1068, 1043, 955, 942, 819.

***MP***: 188.3-189.3 °C.

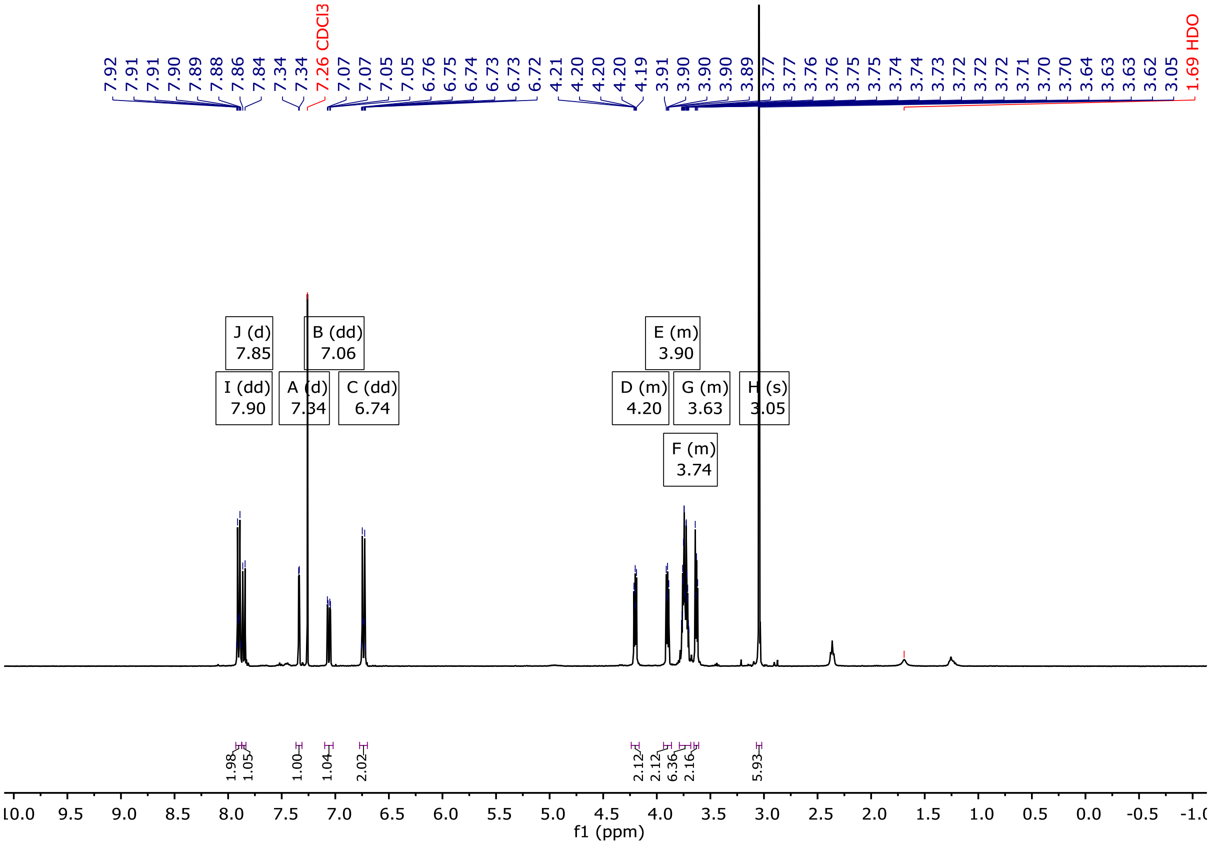

**Figure S18**. $^{1}H$ NMR (400 MHz, $CDCl_3$) spectra of BTA.

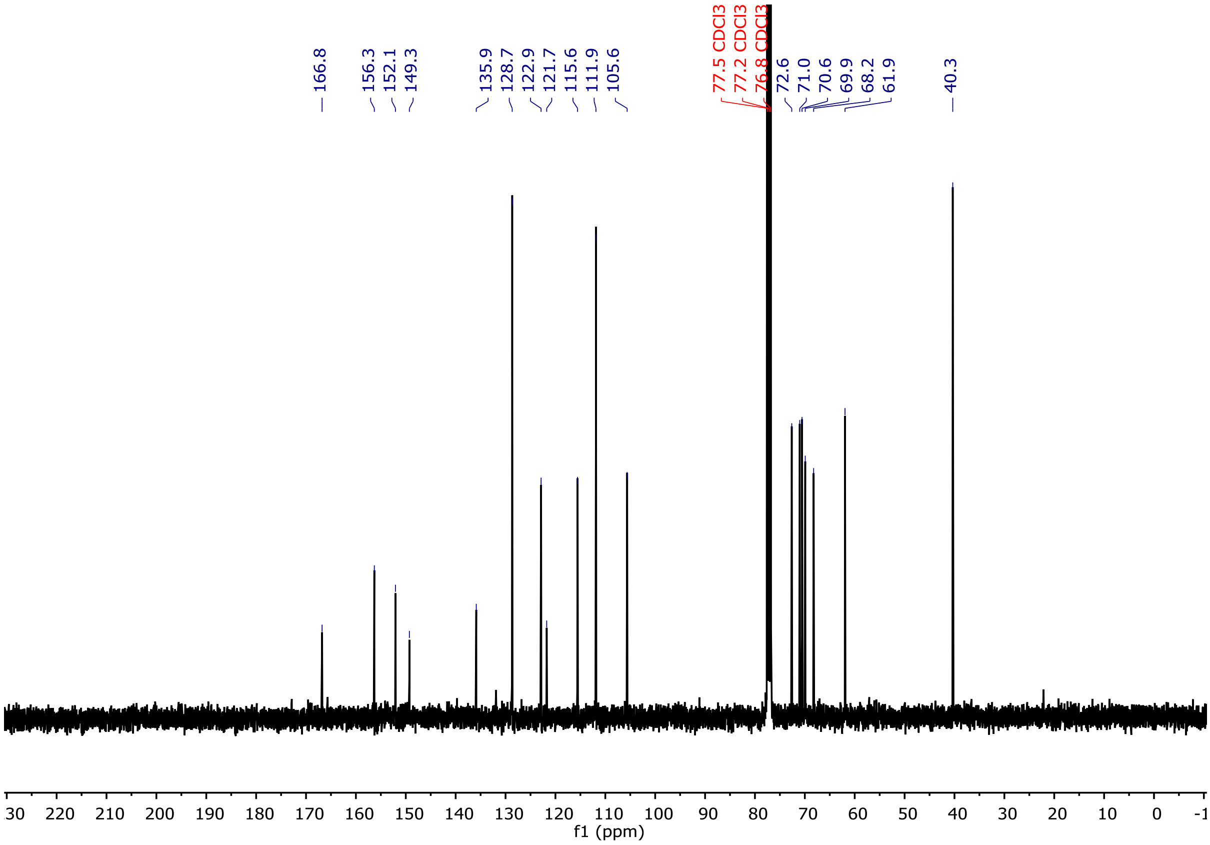

**Figure S19.** $^{13}C$ NMR (101 MHz, $CDCl_3$) spectra of BTA.

## Fluorescence Characterisation

Fluorescence spectral readings were performed on an Agilent Cary Eclipse Fluorescence Spectrophotometer using a scan rate of 600 nm/min, a data interval of 1.0 nm and an averaging time of 0.10 at 25°C. Fluorescence experiments used 20 nm excitation and emission slits and medium PMT voltage, except when specified otherwise. Fluorescence spectra of **BTA** were recorded with 10 nm excitation and emission slits, and low PMT voltage.

Spectral experiments detecting **ThT** used $\lambda_{ex}$ = 440 nm, and measured emissions from $\lambda_{em}$= 470 – 600 nm. Spectral experiments detecting **BTA** used $\lambda_{ex}$ = 360 nm, and measured $\lambda_{em}$= 380 – 600 nm.

Fluorescence anisotropy experiments were performed using 10 nm excitation and emission slits, a scan rate of 120 nm/min, a data interval of 1.0 nm, an averaging time of 0.5 s, and medium PMT voltage at 25 °C. A reference solution of **BTA** in DMSO (2 μM) was used to calculate G-factors. A G-Factor voltage of 425 V was used, and a polarisation/anisotropy voltage of 740 V was used. Anisotropy experiments detecting **BTA** used $\lambda_{ex}$ = 360 nm, and measured $\lambda_{em}$= 433 – 453 nm.

## UV-Visible Characterisation

Stock solutions of ligand in DMSO (10 mM) were diluted into ethanol to obtain a 50 μM solution and placed into a quartz fluorescence cuvette (Hellma Analytics) with a 1 cm pathlength. UV-visible spectra were obtained with an Agilent Cary 60 UV-vis spectrophotometer controlled by Cary WinUV software using a scan rate of 600 nm/min, a data interval of 1.0 nm and an averaging time of 0.10 at 25°C.

## Preparation of αSyn Fibrils

Wild-type human monomeric αSyn in 1xPBS (180 μM) expressed in *E. Coli* and purified as previously reported.[10] The four different morphologies were prepared based on previously reported procedures.[7,8,9]

Three buffers were prepared for aggregating αSyn fibrils. The conditions used were based on literature procedures that appeared to generate fibrils with different morphologies.

Buffer 1: 1xPBS (pH 7.4)[7]

Buffer 2: Tris·HCl (50 mM, pH 7.5) and NaCl (100 mM)[8]

Buffer 3: MES (20 mM, pH 6.1)[9]

A solution of monomeric αSyn in 1xPBS (180 μM) was added to an Amicon Ultra-15 Centrifugal filter (15 kDa MWCO) and centrifuged (15 min, 4000 x g). The retained monomeric αSyn was washed with one of the three buffers to be used for aggregation by adding 5 mL of buffer and centrifuging (15 min, 4000 x g). This wash step was repeated four times in total. The retained filtrate was diluted to 1 mL using the desired buffer and incubated at 37 °C for 72 h with gentle agitation by a magnetic stir bar in an Eppendorf LoBind microcentrifuge tube (2.0 mL). The resultant fibrils were then pelletised in a centrifuge (15 min, 4000 x g), the supernatant removed, and the fibrils gently resuspended in the desired buffer. The absorbance of monomer in the removed supernatant was measured at 280 nm ($\varepsilon = 5{,}960\ M^{-1}\ cm^{-1}$) to determine the concentration of fibrils, given as the concentrated of aggregated monomer (αSyn 1 [Buffer 1]: 109 μM, αSyn 2 [Buffer 2]: 42 μM, αSyn 3 [Buffer 3]: 123 μM).

The αSyn 1s fibrils were prepared by sonicating a solution of αSyn 1 (183 μM) in 1xPBS between 20 and 30 seconds using a probe sonicator (Bandelin, Sonopuls HD 2070), using 10% maximum power and 50% cycles three times. The sample was then separated into aliquots and stored at -21 °C for 6 months until required.

## Biophysical Characterisation of Amyloid Fibrils

*Circular Dichroism Spectra*

Circular dichroism (CD) spectra of αSyn fibrils (1.0 μM) in 1xPBS (pH 7.4) were recorded with a Chirascan CD1 Spectrometer (Applied Photonics Ltd.) equipped with a Series 800 Temperature Controller (Alpha Omega Instruments). Far-ultraviolet measurements (190-250 nm) were recorded at 25 °C with a 1.0 cm optical pathlength, a time-per-point of 1.0 s, a 1.0 nm bandwidth, and a wavelength step of 0.1 nm. CD spectra were averaged over six scans. Data were baseline corrected by subtracting the complete buffer spectrum of 1xPBS (pH 7.4) averaged over six scans. Applied Photophysics Pro-Data Chirascan software was used to smooth the data using Savitsky-Golay smoothing and a window size of eight, and the data was converted to molar ellipticity.

*Transmission Electron Microscopy*

Nanoscale morphologies of fibril samples were observed by transmission electron microscopy (TEM) using a Thermo Scientific (FEI Company) Talos F200X G2 microscope operating at 200 kV. Images were recorded with a Ceta 4k x 4k CMOS camera. For sample preparation, TEM grids (continuous carbon film on 300 mesh Cu) were glow discharged using a Quorom Technologies GloQube at 25 mA for 60 s. A 2 μL sample of fibril in 1xPBS (1.0 μM) was placed on a freshly glow-discharged grid, and after 1.0 min was carefully removed by blotting with filter paper. The sample was negatively stained using 2.0 μL of 2% (w/v) uranyl acetate solution in ethanol for 30 s. The grid was blotted and dried in air for 10 min at room temperature before use.

## In Vitro Binding Assays

*General Procedure for Fluorescence Titrations*

Fluorescence spectra were measured using the general procedure described above for fluorescence characterisation. Stock solutions of ligand in DMSO at concentrations of 1.0 mM were prepared. Stock solutions of αSyn fibrils in 1xPBS (pH 7.4, 10 μM) were prepared. Titration solutions were prepared by diluting ligands (1.0 mM in DMSO) and αSyn fibrils (10 μM in 1xPBS) in 1xPBS (pH 7.4) to the desired concentration. All titrations were performed in 1xPBS (pH 7.4) at 25°C. All titrations were performed alongside a corresponding dilution series in the absence of any amyloid fibrils. Titrations were repeated with at least three independent replicates. Each replicate was performed using titration solutions freshly prepared from different stock solutions. Spectral experiments detecting **ThT** used $\lambda_{ex}$ = 440 nm, and measured emissions from $\lambda_{em}$= 470 – 600 nm. Spectral experiments detecting **BTA** used $\lambda_{ex}$ = 360 nm, and measured $\lambda_{em}$= 380 – 600 nm.

*Saturation Binding Assays*

For saturation binding assays, solutions of ligand (10 μM) and αSyn fibril (500 nM) in 1xPBS (pH 7.4) were titrated into a solution of αSyn fibril (500 nM) in 1xPBS (pH 7.4) at 298 K.

*Fluorescence Anisotropy Binding Assays*

For fluorescence anisotropy assays, solutions of BTA (10 μM) and αSyn fibrils (500 nM) in 1xPBS (pH 7.4) were titrated into a solution of αSyn fibrils (500 nM) in 1xPBS (pH 7.4) at 298 K.

*One-Step Competition Binding Assays*

Solutions of competing ligand L1 (10-50 μM), ThT (1.0 μM), and αSyn fibrils (500 nM) were titrated into a solution of ThT (1.0 μM) and αSyn fibrils (500 nM) in 1xPBS (pH 7.4) at 298 K.

*One-Step Blocked Binding Assays*

Solutions of ligand L0 (S5H: 1.82 μM, OXI: 1.42 μM, ThR: 5.00 μM), ThT (20.0 μM), and αSyn fibrils (500 nM) were titrated into a solution of competing ligand L0 (S5H: 1.82 μM, OXI: 1.42 μM, ThR: 5.00 μM) and αSyn fibrils (500 nM) in 1xPBS (pH 7.4) at 298 K.

*Two-Step Competition Binding Assays*

A standard competition binding assay was first performed as above. Then, a solution of competing ligand L2 (10-50 μM), ligand L1 (S5H: 1.38 μM, OXI: 2.38 μM), ThT (1.0 μM), and αSyn fibrils (500

nM) were titrated into a solution of ligand L1 (S5H: 1.38 μM, OXI: 2.38 μM), ThT (1.0 μM) and αSyn fibrils (500 nM) in 1xPBS (pH 7.4) at 298 K.

*Data fitting*

Fluorescence spectra were analysed using a Microsoft Excel spreadsheet prepared by Professor Christopher Hunter.

The Microsoft Excel spreadsheet fitted the measured fluorescence intensity at a fixed wavelength to a 1:1 binding isotherm using purpose-written VBA macros employing two algorithms, COGS and Simplex. This spreadsheet is generalisable to multiple ligands and binding sites. For a ligand $L$ and binding site $S$, the intensity of the fluorescence emission ($I$) is given by Equation 1,

$$I = \epsilon_f \Phi_f [\mathrm{L}] + \epsilon_b \Phi_b [\mathrm{L} \cdot \mathrm{S}], \qquad \text{Eq. 1}$$

where $\epsilon_f \phi_f$ and $\epsilon_b \phi_b$ are the product of the UV-vis absorption extinction coefficient and the fluorescence quantum yield for free and bound $L$ respectively, [L] is the concentration of free $L$, and $[\mathrm{L} \cdot \mathrm{S}]$ is the concentration of $L$ bound to S. The quantity $\epsilon_{i,f} \Phi_{i,f}$ was measured using dilution series in the absence of host.

Equation 2 is used to fit anisotropy data,

$$r = r_f \frac{[\mathrm{L}]}{[L_{tot}]} + r_b \frac{[\mathrm{L \cdot S}]}{[L_{tot}]}, \qquad \text{Eq. 2}$$

where $r$ is the measured anisotropy of the system, $r_f$ is the anisotropy of the free ligand, and $r_b$ is the anisotropy of the bound ligand.

The concentration of $L$ bound to $S$ is then given by Equation 3,

$$K_d [\mathrm{L} \cdot \mathrm{S}] = [\mathrm{L}][\mathrm{S}], \qquad \text{Eq. 3}$$

where [S] is the concentration of unbound site $S$, and $K_d$ is the dissociation constant of $L$ binding to $S$. The total concentration of $L$, $[\mathrm{L_{tot}}]$, is then given by Equation 4,

$$[\mathrm{L_{tot}}] = [\mathrm{L} \cdot \mathrm{S}] + [\mathrm{L}]. \qquad \text{Eq. 4}$$

The spreadsheet is generalisable to multiple ligands and binding sites, which is required for competition binding assays. The total concentration of binding site, $[L_{tot}]$, is also optimised using this method to avoid assumptions about the stoichiometry of binding sites to protein concentration. For competition binding assays the reporting ligand (ThT) was assumed to bind to two binding sites with an identical dissociation constant to form complexes with an identical optical brightness. For two-step competition binding assays, the fitting procedure assumed that no competition occurred between the first and second competing ligands. This assumption was justified by the fact that the second competing ligand would preferentially displace ThT, which in all instances had the weaker dissociation constant.

## Photophysical Characterisation

*UV-Visible Characterisation*

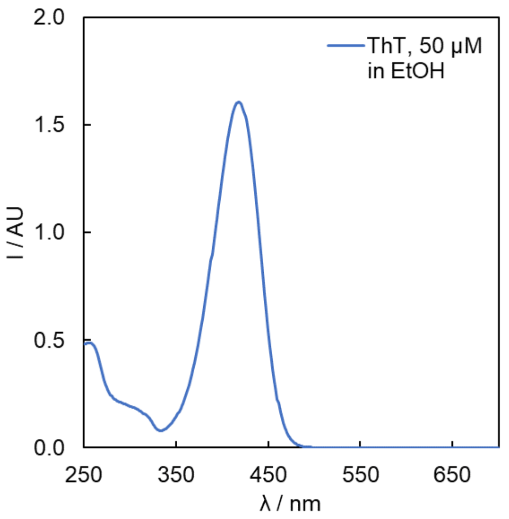


**Figure S20.** UV-vis spectrum of ThT (50 μM) in EtOH at 298 K, with $\lambda_{max}$ = 418 nm.

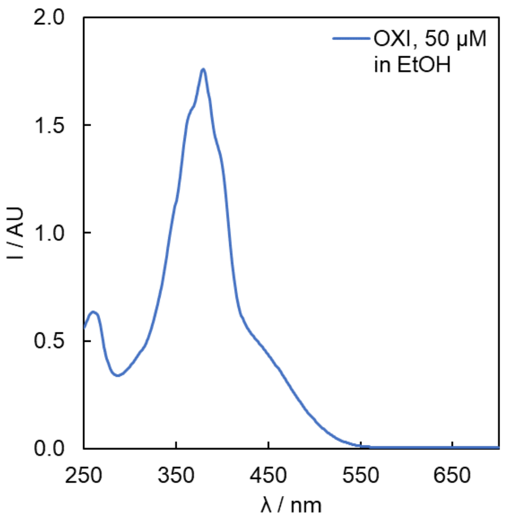


**Figure S21.** UV-vis spectrum of OXI (50 μM) in EtOH at 298 K, with $\lambda_{max}$ = 380 nm.

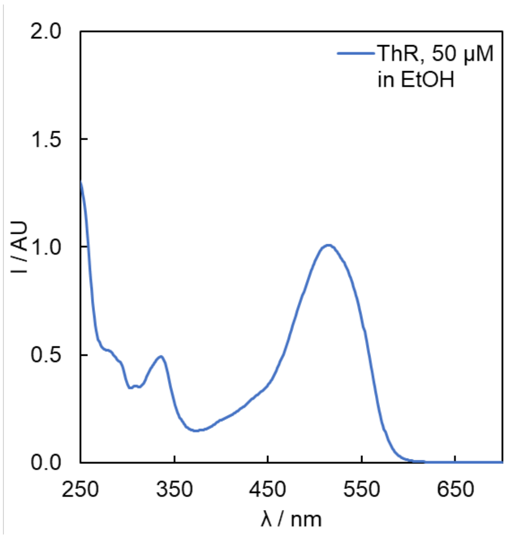


**Figure S22.** UV-vis spectrum of ThR (50 µM) in EtOH at 298 K, with $\lambda_{max}$ = 515 nm.

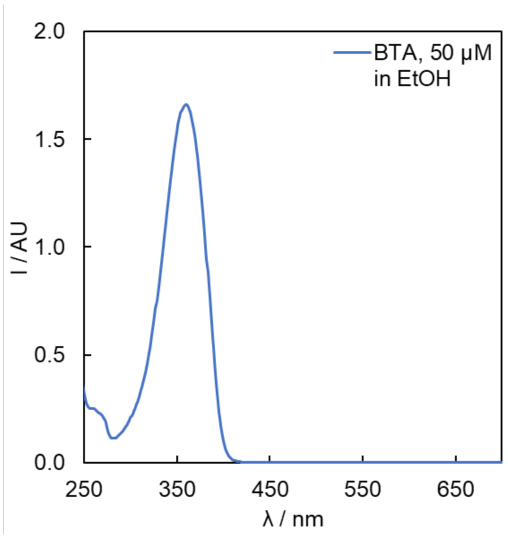


**Figure S23.** UV-vis spectrum of BTA (50 µM) in EtOH at 298 K, with $\lambda_{max}$ = 359 nm.

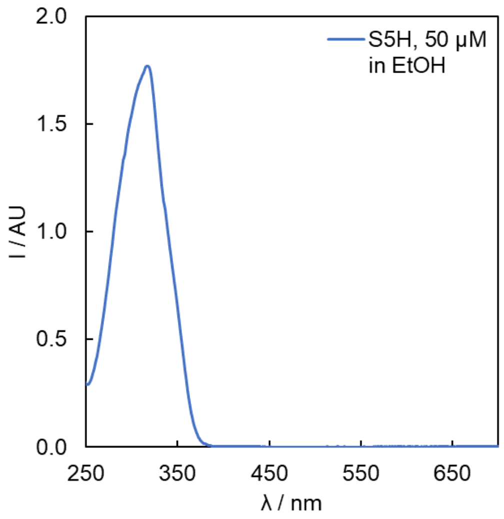


**Figure S24.** UV-vis spectrum of S5H (50 µM) in EtOH at 298 K, with $\lambda_{max}$ = 318 nm.

**Table S1.** Absorbance maxima of ligands (50 µM) in EtOH at 298 K.

| Ligand | $\lambda_{max}$ / nm |
|---|---|
| ThT | 418 |
| OXI | 380 |
| ThR | 515 |
| BTA | 359 |
| S5H | 318 |

*Fluorescence Characterisation*

Fluorescence spectra were obtained according to general methods for each ligand in ethanol (50 μM), in 1xPBS (pH 7.4), and in 1xPBS (pH 7.4) bound to amyloid fibril. Fluorescence emission spectra of ligands were also recorded at the excitation wavelengths of the primary fluorescent ligands used (ThT: $\lambda_{ex}$ = 440 nm, BTA: $\lambda_{ex}$ = 360 nm).

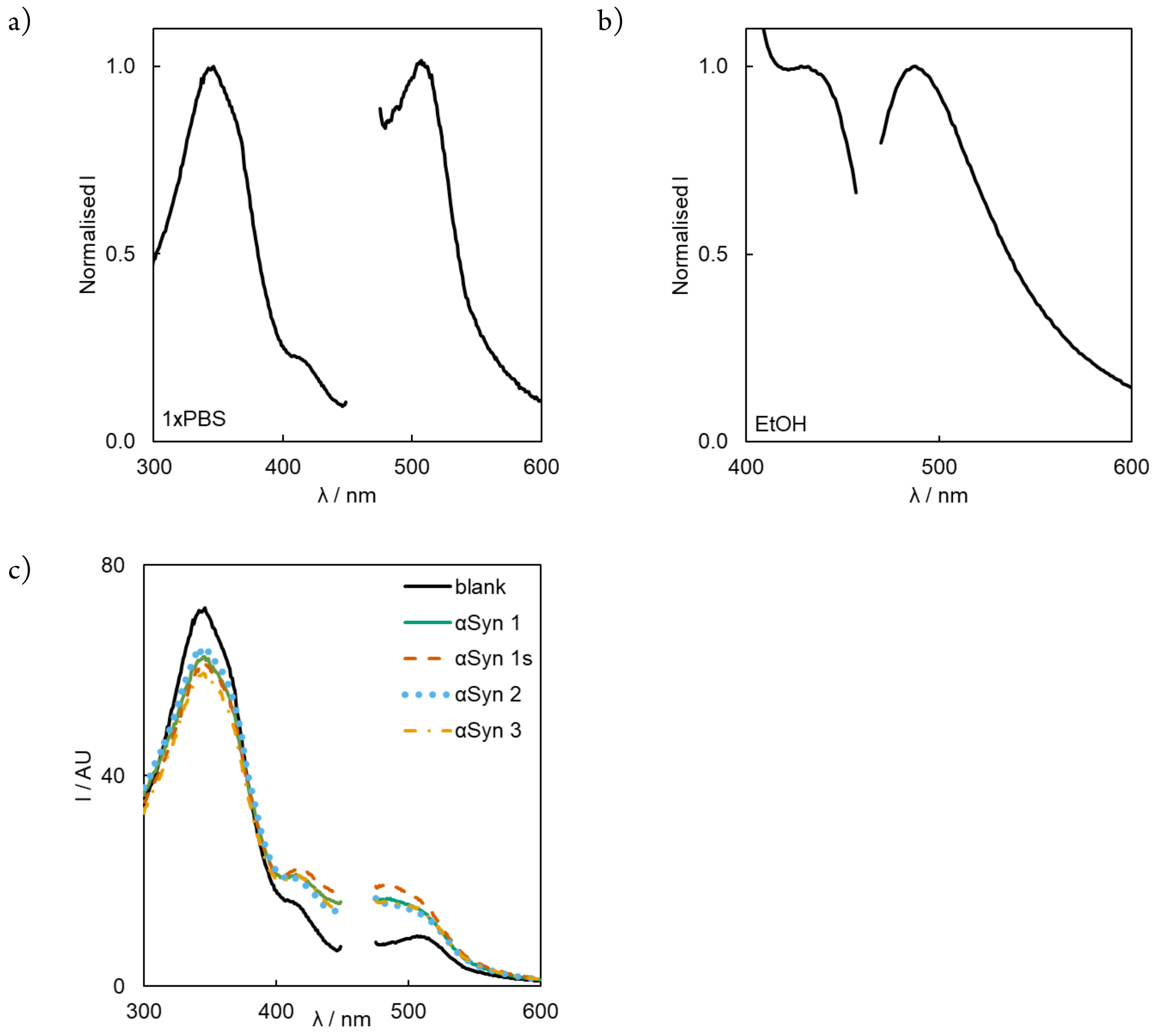


**Figure S25.** Fluorescence spectra of ThT in (a) 1xPBS (pH 7.4, 2.0 μM, $\lambda_{ex}$ = 440 nm, $\lambda_{em}$ = 485 nm, 298 K); (b) EtOH (50 μM, $\lambda_{ex}$ = 440 nm, $\lambda_{em}$ = 487 nm, 298 K); (c) 1xPBS (pH 7.4, 2.0 μM, $\lambda_{ex}$ = 440 nm, $\lambda_{em}$ = 485 nm, 298 K) and in the presence and absence of αSyn fibril preparations (500 nM).

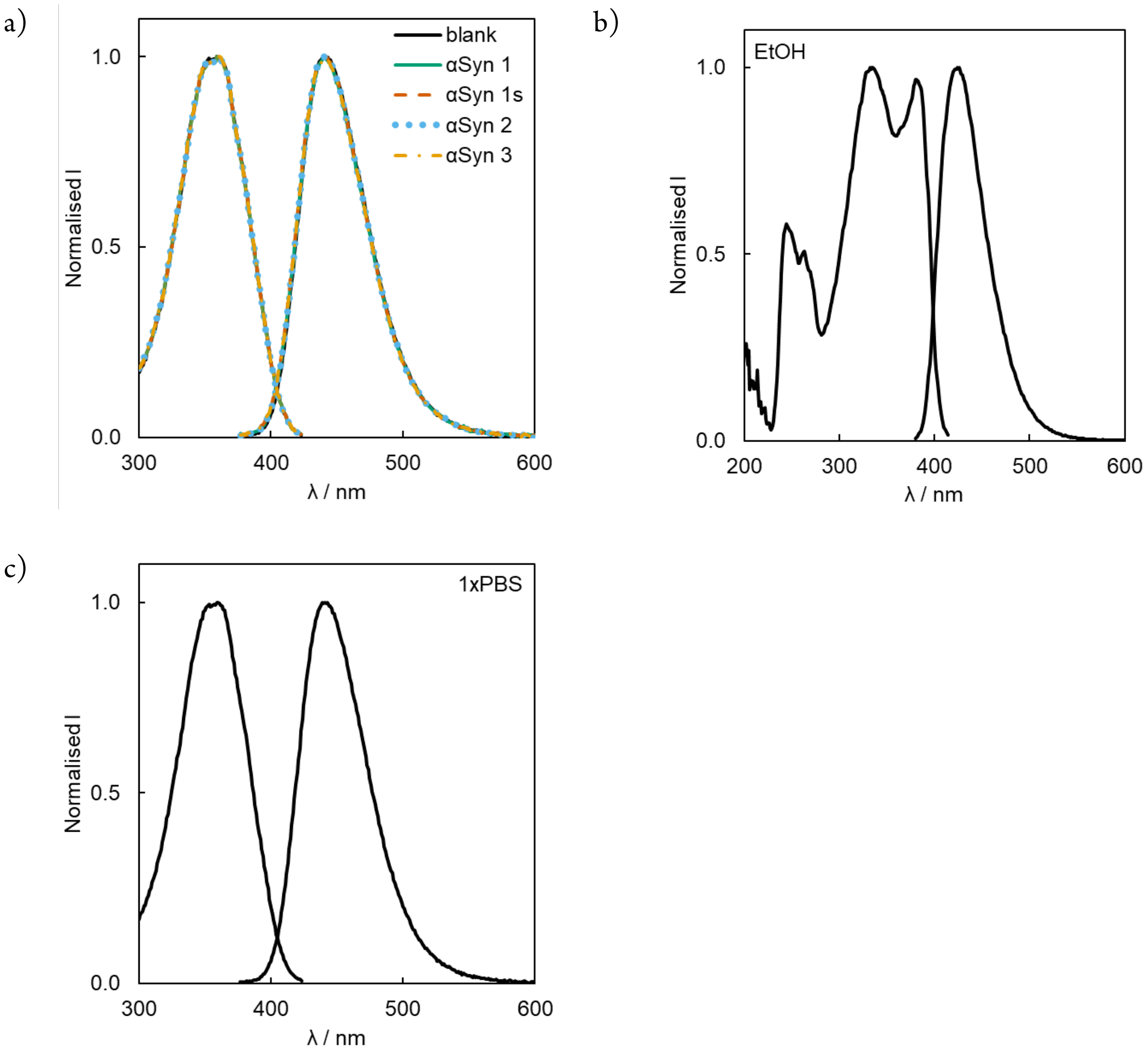


**Figure S26.** Fluorescence spectra of BTA in (a) 1xPBS (pH 7.4, 2.0 μM, $\lambda_{ex}$ = 357 nm, $\lambda_{em}$ = 443 nm, 298 K); (b) EtOH (50 μM, $\lambda_{ex}$ = 360 nm, $\lambda_{em}$ = 424 nm, 298 K); (c) 1xPBS (pH 7.4, 2.0 μM, $\lambda_{ex}$ = 357 nm, $\lambda_{em}$ = 443 nm, 298 K) and in the presence and absence of αSyn fibril preparations (500 nM).

**Table S2.** Emission and excitation maxima for ThT and BTA in 1xPBS or EtOH at 298 K.

| | $\lambda_{ex,\ 1xPBS}$ / nm | $\lambda_{em,\ 1xPBS}$ / nm | $\lambda_{ex,\ EtOH}$ / nm | $\lambda_{em,\ EtOH}$ / nm |
|---|---|---|---|---|
| ThT | 346 | 506 | 430 | 487 |
| BTA | 353 | 441 | 334 | 425 |

## Dilution Series

Dilution series of ligands were performed according to the general methods described.

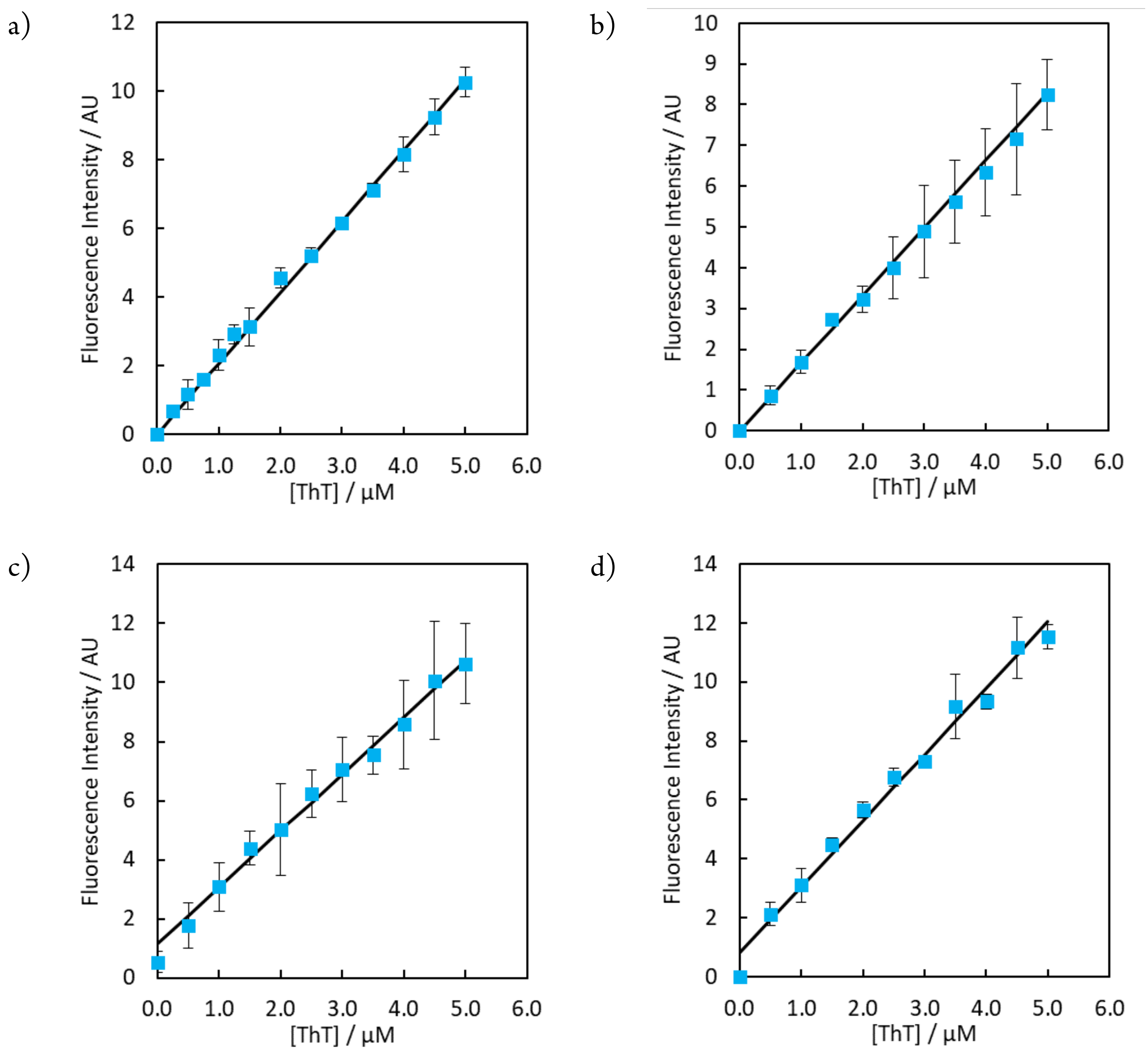


**Figure S27:** Dilution series of ThT into (a) 1xPBS (pH 7.4, 25 °C), showing a line of best fit with a slope of $(\mathbf{1.9} \pm \mathbf{0.2}) \times \mathbf{10^{6}}\ \boldsymbol{M^{-1}}$ and a y-intercept of 0; (b) ThR (2.0 μM) in 1xPBS (pH 7.4, 25 °C), showing a line of best fit with a slope of $(\mathbf{1.7} \pm \mathbf{0.2}) \times \mathbf{10^{6}}\ \boldsymbol{M^{-1}}$ and a y-intercept of 0; (c) OXI (2.0 μM) in 1xPBS (pH 7.4, 25 °C), showing a line of best fit with a slope of $(\mathbf{1.9} \pm \mathbf{0.4}) \times \mathbf{10^{6}}\ \boldsymbol{M^{-1}}$ and a y-intercept of 1.4 ± 0.6; and (d) S5H (2.0 μM) in 1xPBS (pH 7.4, 25 °C), showing a line of best fit with a slope of $(\mathbf{2.2} \pm \mathbf{0.1}) \times \mathbf{10^{6}}\ \boldsymbol{M^{-1}}$ and a y-intercept of 0.8 ± 0.3. Spectra were recorded using $\lambda_{ex}$ = 440 nm and monitoring emission at $\lambda_{em}$ = 483 nm. The experimental measurements are shown as points (error bars represent the 95% confidence interval calculated from at least three independent experiments).

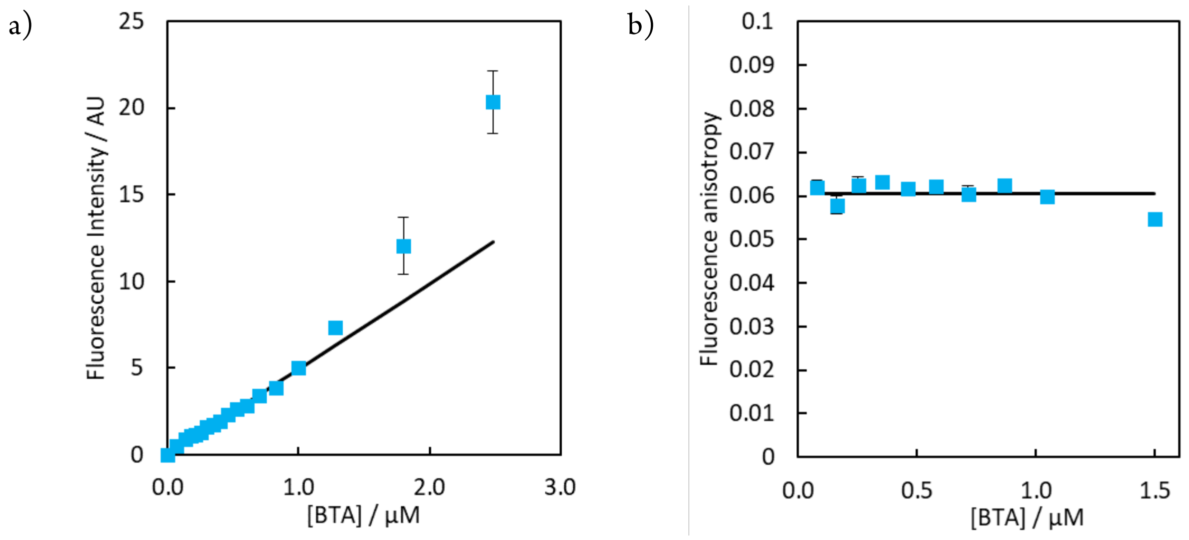


**Figure S28.** (a) Dilution series of BTA into 1xPBS (pH 7.4, 25 °C), showing a line of best fit with a slope of $(\mathbf{4.9 \pm 0.2}) \times \mathbf{10^{6}}\ \boldsymbol{M^{-1}}$ and a y-intercept of 0. Spectra were recorded using $\lambda_{ex}$ = 360 nm and monitoring emission at $\lambda_{em}$ = 443 nm. (b) Fluorescence anisotropy dilution series of BTA into 1xPBS (pH 7.4, 25 °C). Fluorescence anisotropy measurements were recorded using $\lambda_{ex}$ = 360 nm and monitoring emission at $\lambda_{em}$ = 443 nm. The average anisotropy value of the free ligand was 0.061 ± 0.001. The experimental measurements are shown as points (error bars represent the 95% confidence interval calculated from at least three independent experiments).

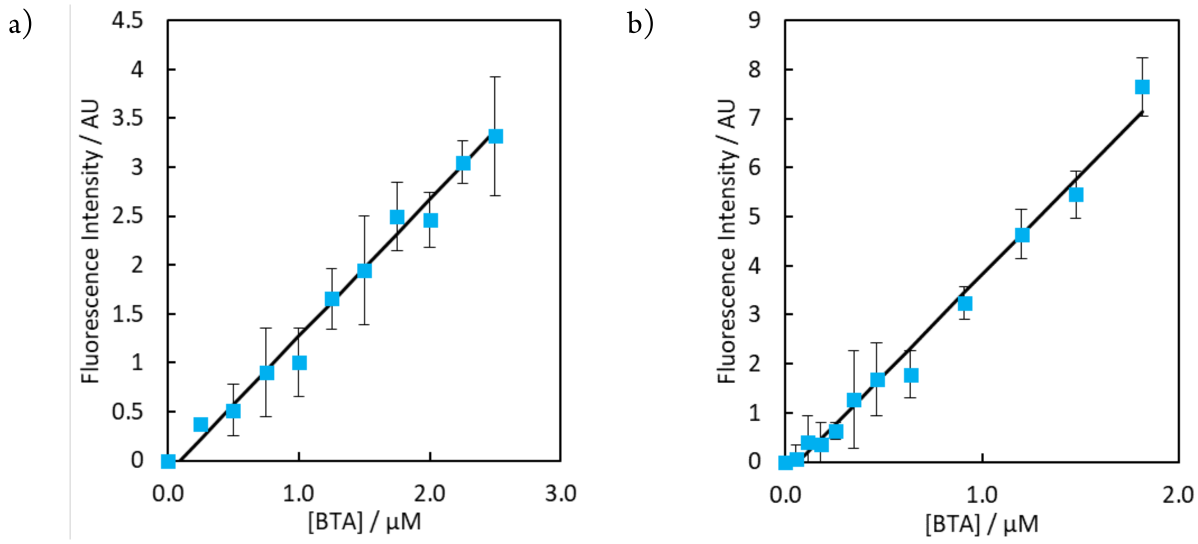


**Figure S29.** Dilution series of BTA into (a) ThT (1.0 μM) in 1xPBS (pH 7.4, 25 °C), showing a line of best fit with a slope of $(\mathbf{1.4 \pm 0.1}) \times \mathbf{10^6}\ \boldsymbol{M^{-1}}$ and a y-intercept of -0.1 ± 0.2; (b) OXI (2.38 μM) and ThT (1.0 μM) in 1xPBS (pH 7.4, 25 °C), showing a line of best fit with a slope of $(\mathbf{4.1 \pm 0.3}) \times \mathbf{10^6}\ \boldsymbol{M^{-1}}$ and a y-intercept of -0.3 ± 0.4. Spectra were recorded using $\lambda_{ex}$ = 440 nm and monitoring emission at $\lambda_{em}$ = 483 nm. The experimental measurements are shown as points (error bars represent the 95% confidence interval calculated from at least three independent experiments).

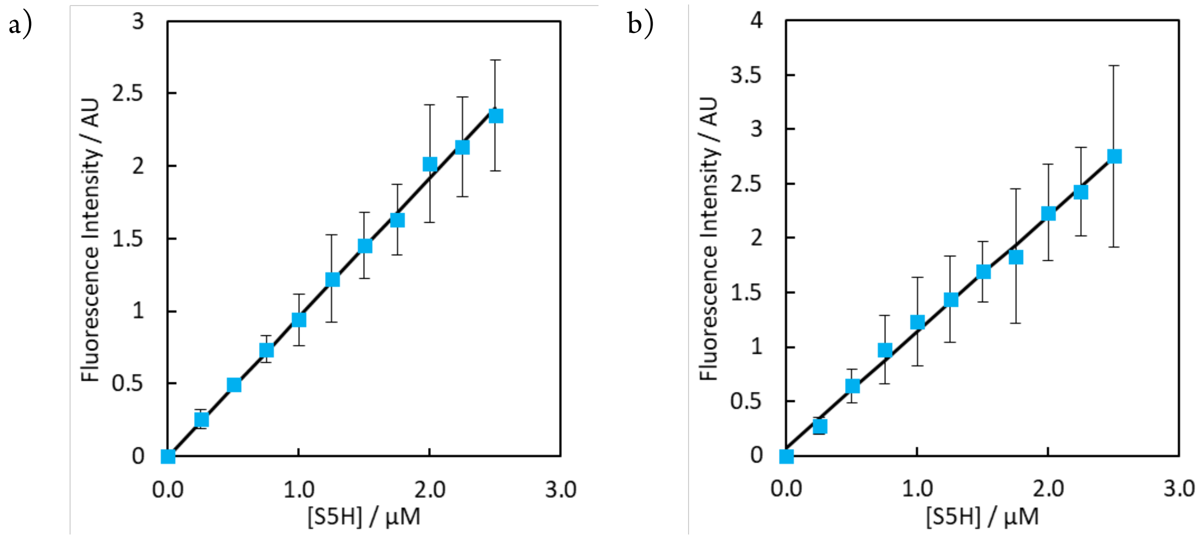


**Figure S30.** Dilution series of S5H into (a) 1xPBS (pH 7.4, 25 °C), showing a line of best fit with a slope of $(\mathbf{9} \pm \mathbf{2}) \times \mathbf{10^5}\ \boldsymbol{M^{-1}}$ and a y-intercept of 0; (b) ThT (1.0 μM) in 1xPBS (pH 7.4, 25 °C), showing a line of best fit with a slope of $(\mathbf{1.1} \pm \mathbf{0.3}) \times \mathbf{10^6}\ \boldsymbol{M^{-1}}$ and a y-intercept of 2.4 ± 0.3. Spectra were recorded using $\lambda_{ex}$ = 440 nm and monitoring emission at $\lambda_{em}$ = 483 nm. The experimental measurements are shown as points (error bars represent the 95% confidence interval calculated from at least three independent experiments).

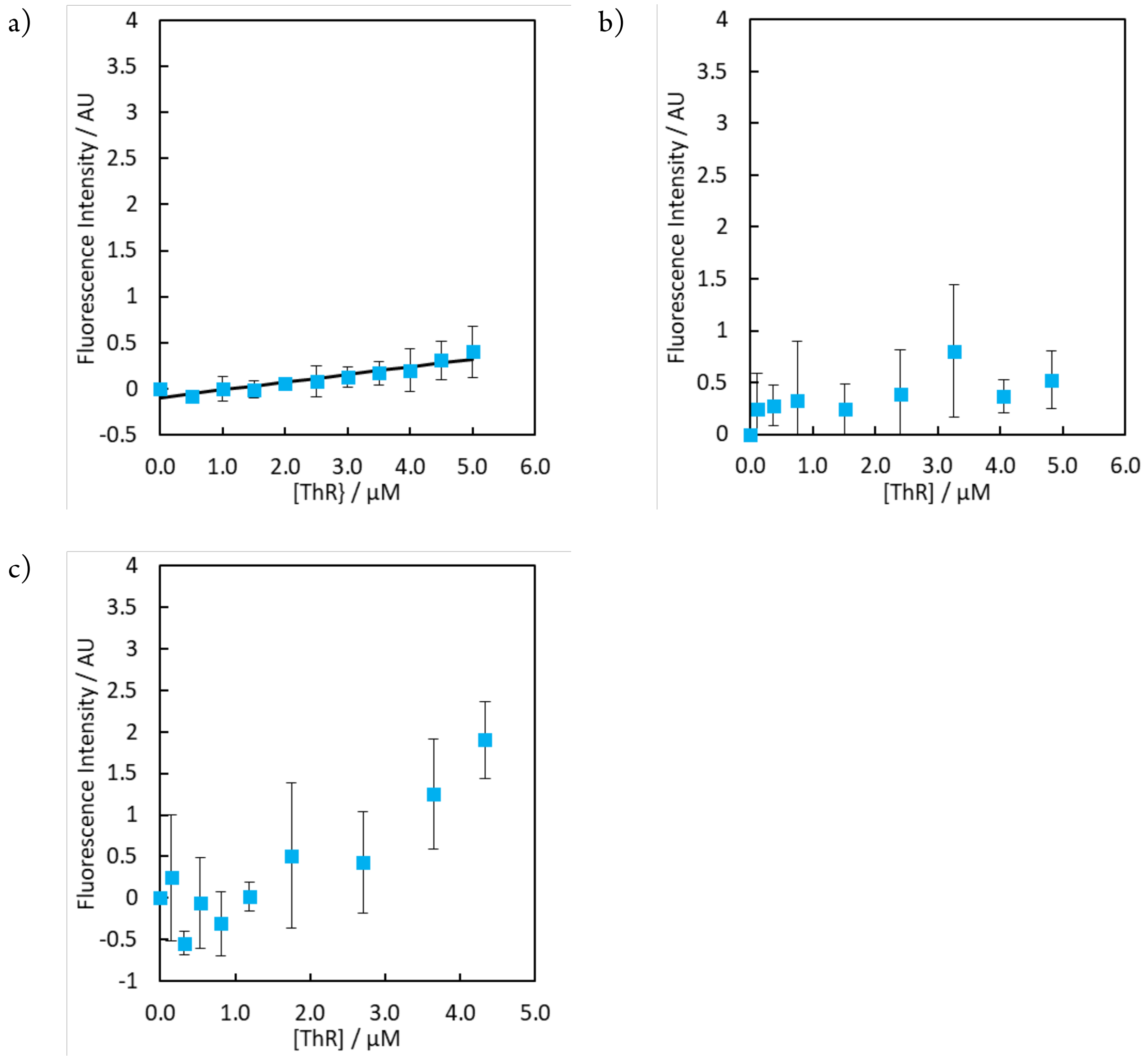


**Figure S31.** Dilution series of ThR into (a) ThT (1.0 µM) in 1xPBS (pH 7.4, 25 °C), showing a line of best fit with a slope of $(\mathbf{8} \pm \mathbf{4}) \times \mathbf{10^4}\ \boldsymbol{M^{-1}}$ and a y-intercept of -0.1 ± 0.1; (b) ThT (1.0 µM) and OXI (2.0 µM) in 1xPBS (pH 7.4, 25 °C); (c) ThT (1.0 µM) and S5H (2.0 µM) in 1xPBS (pH 7.4, 25 °C). Spectra were recorded using $\lambda_{ex}$ = 440 nm and monitoring emission at $\lambda_{em}$ = 483 nm. The experimental measurements are shown as points (error bars represent the 95% confidence interval calculated from at least three independent experiments).

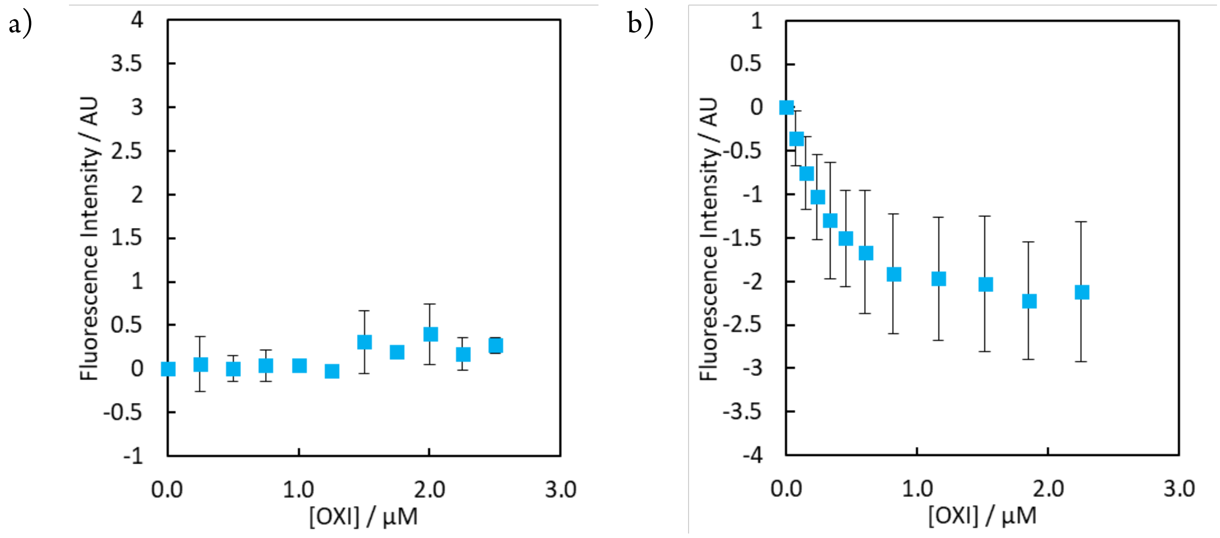


**Figure S32.** Dilution series of OXI into (a) ThT (1.0 µM) in 1xPBS (pH 7.4, 25 °C); (b) into ThT (1.0 µM) and S5H (2.0 µM) in 1xPBS (pH 7.4, 25 °C). Spectra were recorded using $\lambda_{ex}$ = 440 nm and monitoring emission at $\lambda_{em}$ = 483 nm. The experimental measurements are shown as points (error bars represent the 95% confidence interval calculated from at least three independent experiments).

## One-Step Blocked Binding Assays

Binding assays of ThT were performed in the presence of ThR, OXI, and S5H according to the general methods. The presence of these competing ligands influences what sites the binding assays report on. For example, on αSyn 1 fibrils, OXI occupies sites B and C. Therefore, ThT will preferentially bind to the unoccupied site A in the presence of the nanomolar ligand OXI.

The calculated ThT binding constants were all very similar (-log($K_d$/M) = 5.5–6.1). However, the binding of ThT to αSyn 3 was relatively strong in the absence of any other ligands (log($K_d$/M) = 6.7). This titration reported on the binding of ThT to sites A, D, E, and F. Titrations in the presence of OXI and S5H show weaker binding and reported on sites A and D, and D and E respectively. ThT therefore has a higher than expected affinity for Site F on αSyn 3.

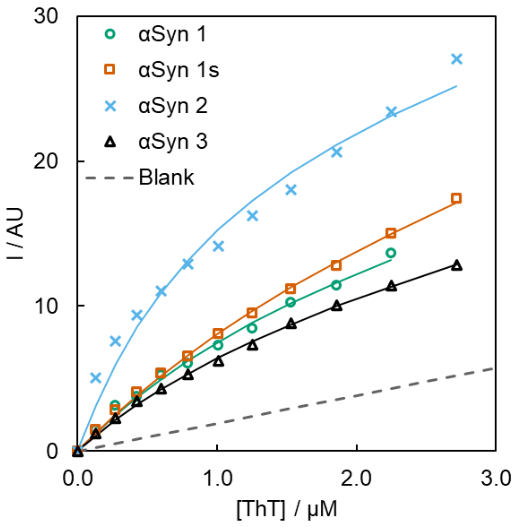


**Figure S33**. Titration of ThT into OXI (1.42 μM) in 1xPBS (pH 7.4, 25 °C). Spectra were recorded using $\lambda_{ex}$ = 440 nm and monitoring emission at $\lambda_{em}$ = 483 nm. Spectra were recorded using $\lambda_{ex}$ = 440 nm and emission intensity was averaged over $\lambda_{em}$ = 481–485 nm. The experimental measurements are shown as points (at least three independent experiments were performed; errors bars are omitted for clarity), and the lines are the best fit to a 1:1 binding isotherm averaged over each independent experiments (αSyn 1: green circles, αSyn 1s: orange squares, αSyn 2: blue crosses, αSyn 3: black triangles).

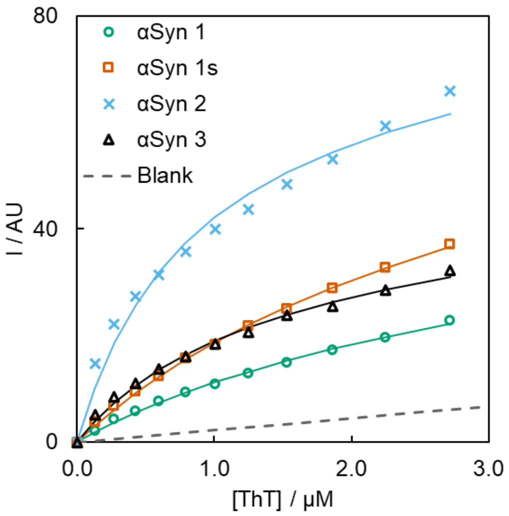


**Figure S34:** Titration of ThT into S5H (1.42 μM) in 1xPBS (pH 7.4, 25 °C). Spectra were recorded using $\lambda_{ex}$ = 440 nm and monitoring emission at $\lambda_{em}$ = 483 nm. Spectra were recorded using $\lambda_{ex}$ = 440 nm and emission intensity was averaged over $\lambda_{em}$ = 481–485 nm. The experimental measurements are shown as points (at least three independent experiments were performed; errors bars are omitted for clarity), and the lines are the best fit to a 1:1 binding isotherm averaged over each independent experiments (αSyn 1: green circles, αSyn 1s: orange squares, αSyn 2: blue crosses, αSyn 3: black triangles).

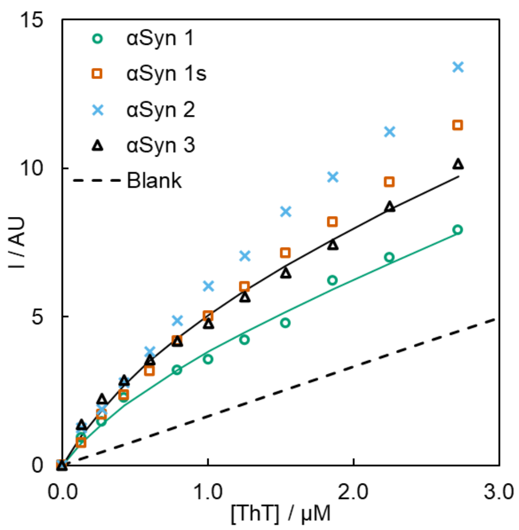


**Figure S35.** Titration of ThT into ThR (5.0 μM) in 1xPBS (pH 7.4, 25 °C). Spectra were recorded using $\lambda_{ex}$ = 440 nm and monitoring emission at $\lambda_{em}$ = 483 nm. Spectra were recorded using $\lambda_{ex}$ = 440 nm and emission intensity was averaged over $\lambda_{em}$ = 481–485 nm. The experimental measurements are shown as points (at least three independent experiments were performed; errors bars are omitted for clarity), and the lines are the best fit to a 1:1 binding isotherm averaged over each independent experiments (αSyn 1: green circles, αSyn 1s: orange squares, αSyn 2: blue crosses, αSyn 3: black triangles). Binding to αSyn 1s and αSyn 2 was too weak to fit.

## Tables of Quantitative Binding Measurements

Tables showing the binding constants for different ligands to different fibrils measured by each binding assay performed. The proposed binding sites targeted by each assay are shown.

**Table S3.** Dissociation constants for binding of ThT to αSyn fibrils.[a]

| | | | Binding Sites | | | | | | |
|---|---|---|---|---|---|---|---|---|---|
| | | | A | A,B | A,B,C | A,D | D,E | A,D,E,F | |
| Binding Site Average | | | 6.0 | 5.7 | 5.8 | 5.7 | 5.8 | 5.8 | |
| **Fibril** | **L0** | **L1** | | | | | | | **Fibril Average** |
| **αSyn 1** | ThT | | - | - | 5.8 ± 0.1 | - | - | - | **5.9** |
| | OXI | ThT | 5.8 ± 0.2 | - | - | - | - | - | |
| | S5H | ThT | - | 5.7 ± 0.2 | - | - | - | - | |
| | ThR | ThT | 6.1 ± 0.2 | - | - | - | - | - | |
| **αSyn 1s** | ThT | | - | - | - | - | - | 5.7 ± 0.1 | **5.6** |
| | OXI | ThT | - | - | - | 5.5 ± 0.1 | - | - | |
| | S5H | ThT | - | - | - | - | 5.6 ± 0.2 | - | |
| | ThR | ThT | n.d. | - | - | - | - | - | |
| **αSyn 2** | ThT | | - | - | - | - | - | 5.9 ± 0.4 | **5.9** |
| | OXI | ThT | - | - | - | 5.8 ± 0.1 | - | - | |
| | S5H | ThT | - | - | - | - | 5.9 ± 0.2 | - | |
| | ThR | ThT | n.d. | - | - | - | - | - | |
| **αSyn 3** | ThT | | - | - | - | - | - | 6.7 ± 0.3 | **5.8** |
| | OXI | ThT | - | - | - | 5.7 ± 0.2 | - | - | |
| | S5H | ThT | - | - | - | - | 5.8 ± 0.1 | - | |
| | ThR | ThT | 6.0 ± 0.1 | - | - | - | - | - | |

[a] "n.d." indicates that the dissociation constant could not be determined from the titration data. "-" indicates that the experiment did not report on this subset of sites. Boxes highlighted in blue indicate outliers, and average values of -log($K_d$/M) exclude these outliers. Errors represent a 99% confidence interval calculated from at least three independent experiments.

**Table S4.** Dissociation constants for binding of BTA to αSyn fibrils.[a]

| | | | | Binding Sites | | |
|---|---|---|---|---|---|---|
| | | | | E,F | E,F,G | |
| Binding Site Average | | | | 6.9 | 7.2 | |
| Fibril | L0 | L1 | L2 | | | Fibril Average |
| αSyn 1 | BTA | | | - | - | - |
| | ThT | BTA | | - | - | |
| | ThT | OXI | BTA | - | - | |
| αSyn 1s | BTA | | | - | 7.8 ± 0.2 | 6.6 |
| | ThT | BTA | | 6.6 ± 0.2 | - | |
| | ThT | OXI | BTA | - | - | |
| αSyn 2 | BTA | | | 7.1 ± 0.1 | - | 7.1 |
| | ThT | BTA | | 7.0 ± 0.1 | - | |
| | ThT | OXI | BTA | - | - | |
| αSyn 3 | BTA | | | - | 7.2 ± 0.1 | 7.2 |
| | ThT | BTA | | 8.0 ± 0.1 | - | |
| | ThT | OXI | BTA | - | - | |

[a] "n.d." indicates that the dissociation constant could not be determined from the titration data. "-" indicates that the experiment did not report on this subset of sites. Boxes highlighted in blue indicate outliers, and average values of -log($K_d$/M) exclude these outliers. Errors represent a 99% confidence interval calculated from at least three independent experiments.

**Table S5.** Dissociation constants for binding of OXI to αSyn fibrils.[a]

| | | | | Binding Sites | | | | |
|---|---|---|---|---|---|---|---|---|
| | | | | B | B,C | E | E,F | |
| Binding Site Average | | | | 7.4 | 7.2 | 6.9 | 7.4 | |
| Fibrils | L0 | L1 | L2 | | | | | Fibril Average |
| αSyn 1 | ThT | OXI | | - | 7.2 ± 0.1 | - | - | 7.3 |
| | ThT | S5H | OXI | 7.4 ± 0.3 | - | - | - | |
| αSyn 1s | ThT | OXI | | - | - | - | 7.4 ± 0.1 | 7.2 |
| | ThT | S5H | OXI | - | - | 7.0 ± 0.4 | - | |
| αSyn 2 | ThT | OXI | | - | - | - | 8.0 ± 0.1 | 6.7 |
| | ThT | S5H | OXI | - | - | 6.7 ± 0.1 | - | |
| αSyn 3 | ThT | OXI | | - | - | - | 8.1 ± 0.1 | 7.1 |
| | ThT | S5H | OXI | - | - | 7.1 ± 0.2 | - | |

[a] "n.d." indicates that the dissociation constant could not be determined from the titration data. "-" indicates that the experiment did not report on this subset of sites. Boxes highlighted in blue indicate outliers, and average values of -log($K_d$/M) exclude these outliers. Errors represent a 99% confidence interval calculated from at least three independent experiments.

**Table S6.** Dissociation constants for binding of S5H to αSyn fibrils.[a]

| | | | Binding Sites | |
|---|---|---|---|---|
| | | | C | F |
| Binding Site Average | | | 6.9 | 6.9 |
| Fibril | L0 | L1 | | |
| αSyn 1 | ThT | S5H | 6.9 ± 0.1 | - |
| αSyn 1s | ThT | S5H | - | 6.7 ± 0.1 |
| αSyn 2 | ThT | S5H | - | 6.7 ± 0.1 |
| αSyn 3 | ThT | S5H | - | 7.3 ± 0.1 |

[a] "n.d." indicates that the dissociation constant could not be determined from the titration data. "-" indicates that the experiment did not report on this subset of sites. Boxes highlighted in blue indicate outliers, and average values of -log($K_d$/M) exclude these outliers. Errors represent a 99% confidence interval calculated from at least three independent experiments.

**Table S7.** Dissociation constants for binding of ThR to αSyn fibrils.[a]

| | | | | Binding Sites | | | | | |
|---|---|---|---|---|---|---|---|---|---|
| | | | | **B** | **B,C** | **D** | **D,E** | **D,E,F** | |
| **Binding Site Average** | | | | **7.2** | **6.9** | **7.0** | **6.8** | **6.7** | |
| **Fibril** | **L0** | **L1** | **L2** | | | | | | **Fibril Average** |
| **αSyn 1** | ThT | ThR | | - | 6.9 ± 0.1 | - | - | - | **7.0** |
| | ThT | S5H | ThR | 7.2 ± 0.1 | - | - | - | - | |
| | ThT | OXI | ThR | - | - | - | - | - | |
| **αSyn 1s** | ThT | ThR | | - | - | - | - | 6.7 ± 0.1 | **6.7** |
| | ThT | S5H | ThR | - | - | - | 6.7 ± 0.2 | - | |
| | ThT | OXI | ThR | - | - | 6.6 ± 0.1 | - | - | |
| **αSyn 2** | ThT | ThR | | - | - | - | - | 6.7 ± 0.1 | **6.8** |
| | ThT | S5H | ThR | - | - | - | 6.8 ± 0.1 | - | |
| | ThT | OXI | ThR | - | - | 6.9 ± 0.3 | - | - | |
| **αSyn 3** | ThT | ThR | | - | - | - | - | 7.3 ± 0.1 | **7.5** |
| | ThT | S5H | ThR | - | - | - | 7.6 ± 0.2 | - | |
| | ThT | OXI | ThR | - | - | 7.4 ± 0.1 | - | - | |

[a] "n.d." indicates that the dissociation constant could not be determined from the titration data. "-" indicates that the experiment did not report on this subset of sites. Boxes highlighted in blue indicate outliers, and average values of -log($K_d$/M) exclude these outliers. Errors represent a 99% confidence interval calculated from at least three independent experiments.

## References

(1) Chu, W.; Zhou, D.; Gaba, V.; Liu, J.; Li, S.; Peng, X.; Xu, J.; Dhavale, D.; Bagchi, D. P.; D'Avignon, A.; Shakerdge, N. B.; Bacskai, B. J.; Tu, Z.; Kotzbauer, P. T.; Mach, R. H. Design, Synthesis, and Characterization of 3-(Benzylidene)Indolin-2-One Derivatives as Ligands for α-Synuclein Fibrils. *J. Med. Chem.* **2015**, *58* (15), 6002–6017.

(2) Lee, Y. H.; Denton, E. H.; Morandi, B. Modular Cyclopentenone Synthesis through the Catalytic Molecular Shuffling of Unsaturated Acid Chlorides and Alkynes. *J. Am. Chem. Soc.* **2020**, *142* (50), 20948–20955.

(3) Hsieh, C. J.; Xu, K.; Lee, I.; Graham, T. J. A.; Tu, Z.; Dhavale, D.; Kotzbauer, P.; Mach, R. H. Chalcones and Five-Membered Heterocyclic Isosteres Bind to Alpha Synuclein Fibrils in Vitro. *ACS Omega* **2018**, *3* (4), 4486–4493.

(4) Qin, L.; Vastl, J.; Gao, J. Highly Sensitive Amyloid Detection Enabled by Thioflavin T Dimers. *Mol. Biosyst.* **2010**, *6* (10), 1791–1795.

(5) Ferrie, J. J.; Lengyel-Zhand, Z.; Janssen, B.; Lougee, M. G.; Giannakoulias, S.; Hsieh, C.-J.; Pagar, V. V.; Weng, C.-C.; Xu, H.; Graham, T. J. A.; Lee, V. M.-Y.; Mach, R. H.; Petersson, E. J. Identification of a Nanomolar Affinity α-Synuclein Fibril Imaging Probe by Ultra-High Throughput in Silico Screening. *Chem. Sci.* **2020**, *11* (7), 12746–12754.

(6) Cui, M.; Ono, M.; Watanabe, H.; Kimura, H.; Liu, B.; Saji, H. Smart Near-Infrared Fluorescence Probes with Donor-Acceptor Structure for in Vivo Detection of β-Amyloid Deposits. *J. Am. Chem. Soc.* **2014**, *136* (9), 3388–3394.

(7) Guerrero-Ferreira, R.; Taylor, N. M. I.; Arteni, A. A.; Kumari, P.; Mona, D.; Ringler, P.; Britschgi, M.; Lauer, M. E.; Makky, A.; Verasdock, J.; Riek, R.; Melki, R.; Meier, B. H.; Böckmann, A.; Bousset, L.; Stahlberg, H. Two New Polymorphic Structures of Human Full-Length Alpha-Synuclein Fibrils Solved by Cryo-Electron Microscopy. *eLife* **2019**, *8*, e48907.

(8) Guerrero-Ferreira, R.; Taylor, N. M. I.; Mona, D.; Ringler, P.; Lauer, M. E.; Riek, R.; Britschgi, M.; Stahlberg, H. Cryo-EM Structure of Alpha-Synuclein Fibrils. *eLife* **2018**, *7*, e36402.

(9) Hoyer, W.; Antony, T.; Cherny, D.; Heim, G.; Jovin, T. M.; Subramaniam, V. Dependence of A-Synuclein Aggregate Morphology on Solution Conditions. *J. Mol. Biol.* **2002**, *322*, 383–393.

(10) Buell, A. K.; Galvagnion, C.; Gaspar, R.; Sparr, E.; Vendruscolo, M., Knowles, T. P. J.; Linse, S.; Dobson, C. M. Solution conditions determine the relative importance of nucleation and growth processes in α-synuclein aggregation. *PNAS,* **2014**, *111,* 7671-7676.
